# Rationale and design of ‘StAtins in Frail oldEr patients with ischemic Stroke or Transient ischemic attack–the Randomized Controlled Trial’ (SAFEST-RCT)

**DOI:** 10.1136/bmjno-2025-001297

**Published:** 2025-10-05

**Authors:** Susanna Rosa Prins, Birgit A Damoiseaux-Volman, Sarah E Vermeer, Patrick M M Bossuyt, Rik Van Eekelen, Judith E Bosmans, Eveline P Van Poelgeest, Fabrice M A C Martens, Marielle H Emmelot-Vonk, Esther Verstraete, Majon Muller, Eric P Moll Van Charante, Michiel Lindhout, Nathalie Van Der Velde, Renske M Van Den Berg-Vos

**Affiliations:** 1 Department of Neurology, Amsterdam UMC Locatie AMC, Amsterdam, Netherlands; 2 Department of Medical Informatics, Amsterdam UMC, Amsterdam, Netherlands; 3 Amsterdam Public Health Research Institute, University of Amsterdam, Amsterdam, Netherlands; 4 Department of Neurology, Rijnstate Hospital, Arnhem, Netherlands; 5 Department of Epidemiology and Data Science, Amsterdam Public Health Research Institute, Amsterdam, Netherlands; 6 Department of Health Sciences, Amsterdam Public Health Research Institute, Amsterdam, Netherlands; 7 Expertise Centre for Pharmacotherapy in Older People (Ephor), Amsterdam, Netherlands; 8 Department of Cardiology, Amsterdam UMC Locatie AMC, Amsterdam, Netherlands; 9 Department of Geriatrics, University Medical Center Utrecht, Utrecht, Netherlands; 10 Departments of Internal Medicine and Geriatrics, Amsterdam UMC, Amsterdam, Netherlands; 11 Department of General Practice, Amsterdam UMC, Amsterdam, Netherlands; 12 Department of Public and Occupational Health, Amsterdam UMC, Amsterdam, Netherlands; 13 Dutch Brain Injury Association (Hersenletsel.nl), Velp, Netherlands; 14 Department of Neurology, OLVG Hospital, Amsterdam, Netherlands

**Keywords:** STROKE, QUALITY OF LIFE, CHOLESTEROL, GERIATRICS

## Abstract

**Introduction:**

Statin therapy is known to reduce subsequent cardiovascular events in patients who had an ischaemic stroke and transient ischaemic attack (TIA). However, its effectiveness and safety in frail older adults with a recent stroke or TIA are uncertain, leading to variations in clinical practice. ‘StAtins in Frail oldEr patients with ischemic Stroke or Transient ischemic attack–the Randomized Controlled Trial’ (SAFEST-RCT) aims to investigate the effectiveness of initiating versus not initiating statin therapy in this vulnerable population, to optimise secondary prevention strategies.

**Methods and analysis:**

This multicentre, prospective, randomised, open-label study aims to enrol 612 frail adults ≥70 years with a recent acute ischaemic stroke or TIA across 22 Dutch hospitals. The study compares prescribing versus not prescribing statins in terms of health-related quality of life, major adverse cardiovascular event-free survival and societal costs over a 2-year follow-up period.

**Ethics and dissemination:**

The SAFEST-RCT protocol was approved by the Ethics Committee of Amsterdam UMC. It complies with the Declaration of Helsinki and is classified as a healthcare evaluation. Recruitment began in March 2025. Results will be published in open access journals, presented at conferences, shared via the Dutch Brain Injury Association and integrated into national guidelines to support implementation in routine care.

**Trial registration number:**

NCT06785727.

**WHAT IS ALREADY KNOWN ON THIS TOPIC**

- Statins reduce cardiovascular events after ischaemic stroke or transient ischaemic attack (TIA), but evidence for their use in frail older adults is lacking due to their under-representation in trials, resulting in clinical uncertainty.

**WHAT THIS STUDY ADDS**

- This trial evaluates the effectiveness of prescribing versus not prescribing statins on quality of life and cardiovascular outcomes in frail older patients who had a stroke and TIA.

**HOW THIS STUDY MIGHT AFFECT RESEARCH, PRACTICE OR POLICY**

- Findings will provide essential guidance for clinical decision-making in this vulnerable and understudied population.

## Introduction

### Background

 Age is a major risk factor for ischaemic stroke and transient ischaemic attack (TIA).^1^ With the global population ageing rapidly, the global incidence of these conditions is expected to rise by 81% between 2021 and 2050.^2^ Ischaemic stroke is a leading cause of disability and generates significant economic burden.^1 3^ As individuals who have suffered an ischaemic stroke or TIA are at increased risk for subsequent cardiovascular events,^1 4 5^ secondary prevention is deemed essential.^6^

Statins are a key component of secondary prevention and have been proven effective in middle-aged patients who had a stroke and TIA.^6 7^ Due to their higher baseline risk, the absolute benefit of statins is expected to be greater in older individuals. However, due to the under-representation of frail older patients in statin trials, there is limited evidence regarding the effectiveness and safety of statins in this population.^8^

A recent meta-analysis of 28 randomised controlled trials (RCTs), including the Pravastatin in elderly individuals at risk of vascular disaese (PROSPER) trial, the Heart Protection Study (HPS) and the Justification for the use of statin in prevention: an intervention trial evaluating rosuvastatin (JUPITER) trial, showed that statins used for secondary prevention lowered the annual incidence of major adverse cardiovascular events (MACE) from 5.8% to 4.8% in people aged 70–75 years, and from 6.8% to 6.0% in people over 75 years.^7^ However, the 17 trials that included older participants excluded individuals with conditions such as multimorbidity, cognitive impairment, life expectancy of less than 5 years, severe heart failure, chronic liver or kidney disease or uncontrolled diabetes. This likely restricted the inclusion of frail individuals. Given that frailty affects approximately 20% of community-dwelling adults aged over 70, 31% of those over 80 and 51% of those over 90 years,^9^ the exclusion of this subgroup creates a substantial evidence gap.

While it might be tempting to extrapolate the benefits of statin therapy from fit to frail older patients, substantial differences between these populations must be acknowledged. In frail older adults, it is essential to consider time to benefit.^10^ Both frailty and a history of ischaemic stroke or TIA independently reduce life expectancy in older individuals, and their combination further worsens prognosis.^11^,^13^ Although lipid lowering and plaque stabilisation can occur within months after starting statin therapy,^14^ a full reduction in cardiovascular events typically requires years.^15^ The results of a meta-analysis indicated that 2.5 years of statin therapy are needed to prevent one MACE per 100 adults aged 50–75.^15^ Consequently, frail older stroke survivors with limited life expectancy may not live long enough to fully benefit from statin therapy.

Frailty also increases the risk of adverse effects.^16^ Statins can cause muscle pain, myopathy, elevated liver enzymes and diabetes mellitus.^17 18^ No significant association has been found between statin use and adverse cognitive effects.^19^ However, the under-representation of frail older individuals in clinical trials may have led to an underestimation of the full spectrum of statin-related risks. Age-related changes in pharmacokinetics and pharmacodynamics, comorbidities and polypharmacy further elevate the risk of adverse effects and drug interactions in this population.^20^,^24^ Even mild adverse effects like myopathy can severely impact mobility, increase fall risk and reduce quality of life (QoL) in frail individuals.^25^ While older adults often prioritise health-related quality of life (HRQoL), daily functioning and independence over mortality,^26 27^ statin trials typically focus primarily on outcomes such as recurrence of cardiovascular events and mortality.^28^ A recent systematic review and meta-analysis by our group found no conclusive evidence that statin use improves QoL or functional status in older patients following ischaemic stroke or TIA.^29^ Furthermore, an RCT involving individuals with a life expectancy of less than 1 year found that discontinuation of statin therapy was associated with improvements in QoL.^30^

Stroke-related healthcare costs are substantial.^3^ Statin therapy as secondary prevention is generally considered cost-effective.^31^ However, in frail older patients with limited life expectancy, higher risk of adverse effects and potentially diminished QoL benefits, cost-effectiveness remains uncertain.

### Objectives

The lack of strong evidence for statin use for secondary prevention in frail older adults has led to uncertainty in clinical guidelines. The 2018 American College of Cardiology guideline recommends statin therapy for patients over 75 who had a stroke, after evaluating recurrence risk, adverse effects, drug interactions and frailty.^32^ Similarly, the 2021 European Society of Cardiology guideline emphasises the need to consider frailty, polypharmacy and muscle symptoms in older adults.^33^ However, neither provides specific guidance for the frail population, contributing to uncertainty and variations in clinical practice.^8^

The StAtins in Frail oldEr patients with ischemic Stroke or Transient ischemic attack–the Randomized Controlled Trial (SAFEST-RCT) aims to clarify these uncertainties by comparing the effects of starting versus withholding statins on HRQoL and MACE-free survival in frail older patients during the first 2 years after ischaemic stroke or TIA.

## Methods

### Study design

This multicentre, prospective, randomised, open-label trial with blinded outcome evaluation is being performed across 22 Dutch hospitals (online supplemental appendix 1: participating study sites). The protocol follows the Standard Protocol Items: Recommendations for Interventional Trials 2013 guideline.^34^

### Participants

Eligible participants must meet the following criteria: (1) a neurologist-confirmed ischaemic stroke or TIA within 6 weeks prior to inclusion; (2) an age ≥70 at the time of the event; (3) not receiving statins at the time of the event; and (4) frailty, defined as a Clinical Frailty Scale (CFS) score of 4–7 pre-event and/or 6–7 post-event.^35^ The CFS evaluates domains such as daily and cognitive functioning and comorbidity, generating a frailty score from 1 (very fit) to 9 (terminally ill); scores ≥4 usually indicate frailty due to increased susceptibility to adverse outcomes, including reduced life expectancy.^35 36^

Exclusion criteria consist of: (1) a history of serious statin-related adverse reactions or contraindications (eg, severe liver disease); (2) very severe frailty (CFS ≥8); (3) inability to communicate in Dutch; (4) inability to respond to questions, independently or with the assistance of a proxy; (5) inability or unwillingness to provide written informed consent, independently or with the assistance of a proxy; (6) extremely high cardiovascular risk, defined as ≥1 cardiovascular event in the year before the index event; and (7) non-atherosclerotic stroke or TIA with no vascular risk factors.

Verbal consent to be contacted by an investigator is obtained by the treating neurologist. The local investigator then provides detailed study information (online supplemental appendix 2: participant information form) and obtains written informed consent, allowing at least 48 hours for consideration. Additional consent is sought for ancillary and follow-up studies. Randomisation is performed using Castor EDC (v.2025.1.2.0). Block (2:4) randomisation is used, stratified by hospital and type of event (ie, TIA or ischaemic stroke), with a 1:1 allocation ratio.

### Intervention

Allocation details are communicated to the treating neurologist. In the intervention group, a statin is newly prescribed. Low-density lipoprotein (LDL) target levels are <1.8 mmol/L (<70 mg/dL) after atherosclerotic ischaemic stroke or TIA, and <2.5 mmol/L (<100 mg/dL) after non-atherosclerotic ischaemic stroke or TIA in the presence of vascular risk factors, based on the Dutch Stroke guideline (May 2019).^37^ Statin type and dose are at the neurologist’s discretion, and the statin is collected by the participants at their own pharmacy. In the control group, no statin is prescribed, in line with the Dutch Cardiovascular Risk Management guideline (March 2019), which recommends considering not to start statin treatment in frail older patients, especially when an adverse effect is being suspected or when life expectancy is reduced.^38^

### Data collection and outcomes

The study evaluates the impact of statin prescription on two primary outcomes:

1. *HRQoL*, measured by the Patient-Reported Outcomes Measurement Information System 10-Question Short Form (PROMIS-10), which includes subscales for mental health and physical health.^39 40^
2. *Three-point MACE-free survival*, defined as survival without any of the following MACEs: cardiovascular death, non-fatal myocardial infarction or non-fatal stroke. MACE events are self-reported by participants or reported by a proxy using a calendar returned every 3 months and are verified annually with treating physicians.

Secondary outcomes include functional outcome assessed using the modified Rankin Scale^41^; falling using a falls calendar; cognitive functioning using the Montreal Cognitive Assessment (MoCA)^42^ or the Telephone version of MoCA^43^; quality-adjusted life-years (QALYs) based on the EuroQol 5-Dimension 5-Level (EQ-5D-5L)^44^ questionnaire; and societal costs using The Older Persons and Informal Caregivers Survey–Minimum DataSet (TOPICS-MDS).^45^

**Figure 1:**
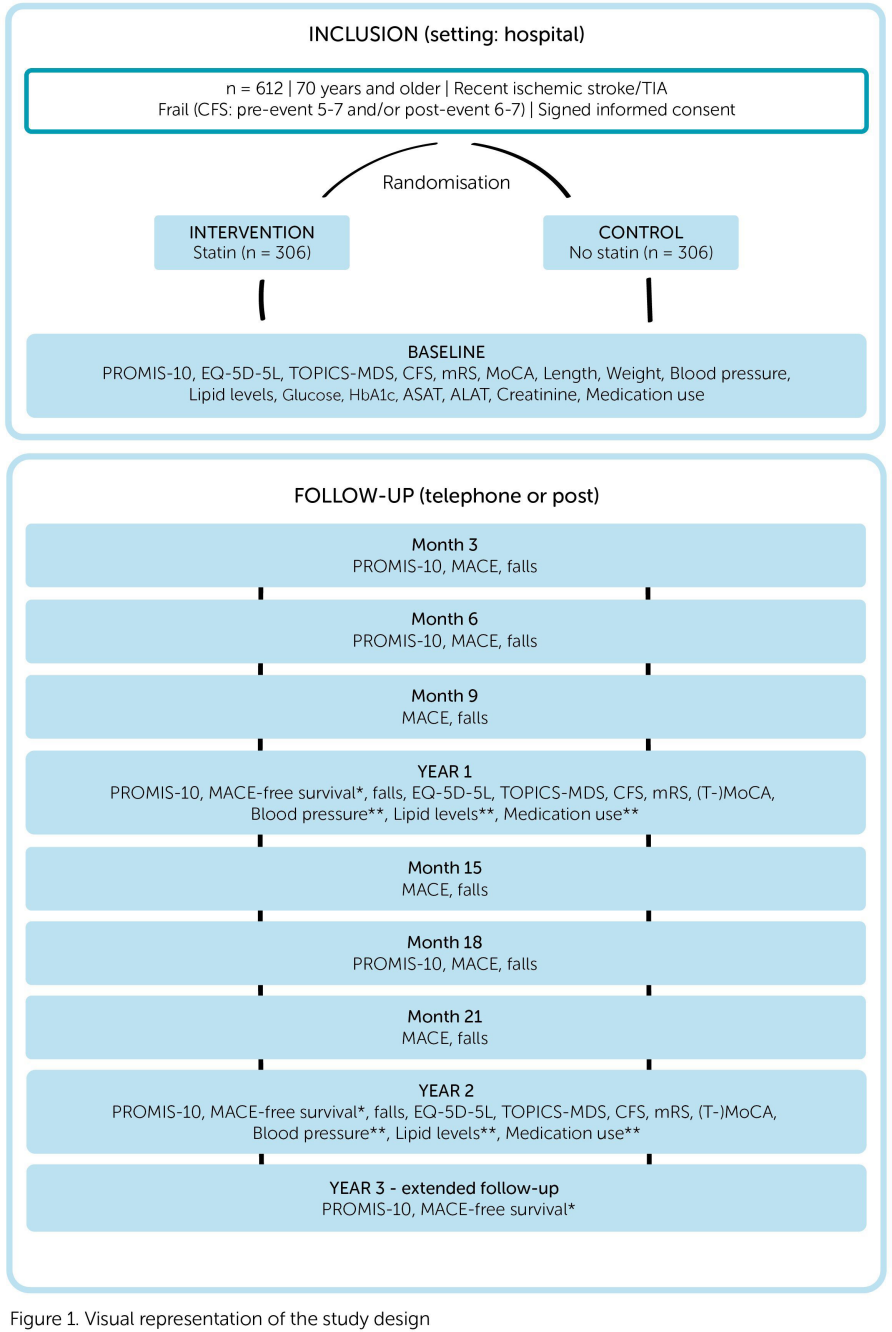
Visual representation of the study design. *Control physician. **Info from physician/pharmacy. CFS, Clinical Frailty Scale; EQ-5D-5L, EuroQol 5-Dimension 5-Level questionnaire; MACE, major adverse cardiovascular event; MoCA, Montreal Cognitive Assessment; ASAT, Aspartate Aminotransferase; ALAT, Alanine Aminotransferase; mRS, modified Rankin Scale; PROMIS-10, Patient-Reported Outcomes Measurement Information System 10-Question Short Form; TIA, transient ischaemic attack; (T-)MoCA, Telephone version of Montreal Cognitive Assessment; TOPICS-MDS, The Older Persons and Informal Caregivers Survey–Minimum DataSet.

At baseline, demographics, medication use and recent clinical metrics and laboratory values are extracted from the electronic patient record by local investigators. Furthermore, the PROMIS-10, MoCA, EQ-5D-5L, TOPICS-MDS and CFS are assessed. Follow-up continues for 24 months, with an optional 1-year extension for early enrollees. Assessment frequencies vary by outcome measure (figure 1). Participants, caregivers and local investigators are not blinded. However, all follow-up is conducted by investigators from the central research team who are blinded to treatment allocation.

Adherence to the intervention is monitored using pharmacy records. To minimise participant burden and enhance engagement, follow-up is conducted by phone or post, with no extra clinic visits or tests. The primary outcome (PROMIS-10) is relatively brief, which facilitates high completion rates. When questionnaires returned by post are incomplete, participants or their proxies are contacted by telephone to obtain the missing information. The study team will make repeated attempts when necessary to ensure that key outcomes are collected as completely as possible. At 12 and 24 months, general practitioners provide the most recent laboratory and blood pressure data available from routine care. Measurements performed within 6 months of the follow-up time point are accepted. Additionally, the SAFEST-RCT is part of the ‘Versneld Evalueren’ (Accelerated Evaluation) pilot by the national programme Care Evaluation and Appropriate Use (‘*Zorgevaluatie en Gepast Gebruik*’), which promotes rapid evaluation and implementation of healthcare innovations.

### Patient and public involvement

This study is supported by the Dutch Brain Injury Association (Hersenletsel.nl). A patient representative from the association was closely involved from the early stages of the study, including the overall study design, and the selection of outcome measures and questionnaires. The burden of the intervention and time investment for participants were jointly assessed with the representative and association members. The association will also assist in disseminating the results through their website and relevant patient channels.

### Sample size

We hypothesise that in frail older patients with recent ischaemic stroke or TIA, starting statins will cause a clinically relevant decrease in HRQoL without substantially improving MACE-free survival. For HRQoL, we expect baseline PROMIS-10 subscale scores of approximately 45, with an SD of 9.0 and a Minimal Clinically Important Difference of 4.5, approximated as 0.5 SD.^46 47^ Frailty and prior ischaemic stroke or TIA both reduce life expectancy in older adults.^11^,^13^ Based on previous studies, we estimate a 2-year survival rate of 52% in the control group.^48^,^50^ Cardiovascular events increase with age, and this older, frail cohort is therefore at higher baseline risk for MACE. Statin use in secondary prevention after ischaemic stroke or TIA in people of all ages has shown reductions in recurrent stroke (HR 0.89), myocardial infarction (HR 0.55) and cardiovascular death (HR 0.78).^51^ For our composite outcome of MACE-free survival in frail older patients, higher baseline risk and limited life expectancy support an HR estimate of 0.70. A total of 612 participants provide 99% power to detect a >4.5-point decrease in HRQoL and 80% power to detect an HR of 0.70 for MACE-free survival, using a two-sided alpha of 0.05, and accounting for 5% loss to follow-up.^52^ Loss to follow-up is expected to be minimal, as most discontinuations will involve study outcomes (MACE or non-cardiovascular death), and partial follow-up still contributes to the time-to-event analysis.

Sample size calculations are based on the Hotelling Lawley Trace test, using a linear exponent autoregressive model with a base correlation of 0.5 and a decay of 0.3.^53^ With approximately 6800 eligible patients annually in the Netherlands based on Dutch health insurance data,^54^ we expect to reach our recruitment target.

### Statistical methods

Primary analyses will follow the intention-to-treat principle. A per-protocol sensitivity analysis will include only participants who adhered to their assigned treatment until censoring. Baseline characteristics will be summarised using means with SDs, medians with IQRs or percentages. Differences at baseline will be tested using analysis of variance or χ^2^ tests.

#### Primary outcomes

HRQoL (PROMIS-10) will be analysed using a mixed model for repeated measures, with deceased patients scored as 0. Group differences over time will be reported with two-sided 95% CIs. MACE-free survival will be analysed using a time-to-event approach, with patients who withdraw early being censored at the time of withdrawal. The effect of statins will be expressed as an HR using a Cox regression model. Adjustments will be made for the type of event (TIA or ischaemic stroke), as these conditions may differ in baseline risk and prognosis. The assumption of proportionality will be tested, and Kaplan-Meier curves will visualise group differences in survival. Exploratory subgroup analyses of the primary outcomes will be performed by age, sex, type of event (ischaemic stroke or TIA), degree of frailty and LDL value.

#### Secondary outcomes

Functional outcome and cognitive functioning will also be analysed using a mixed model for repeated measures. Falls will be assessed with a time-to-event analysis and Kaplan-Meier curves for fall-free survival. Total falls per participant over 2 years will be analysed using a binomial negative regression analysis. Bivariate regression analyses will estimate cost and effect differences. Incremental cost-effectiveness ratios (ICERs) will be calculated by dividing the difference in the mean total costs between the treatment groups by the difference in mean effects on the primary outcomes and QALYs.^55^ Statistical uncertainty surrounding the ICERs will be evaluated using bias-corrected and accelerated bootstrapping with 5000 replications, and presented via cost-effectiveness planes and cost-effectiveness acceptability curves.^56^

## Ethics and dissemination

The protocol was registered on ClinicalTrials.gov (NCT06785727) on 19 December 2025. The study complies with the Declaration of Helsinki and is classified as a healthcare evaluation, comparing two standard-of-care strategies recommended in Dutch guidelines. Given the study’s low-risk nature, a Data Safety Monitoring Board and subject insurance were not required. All centres are covered by institutional liability insurance in accordance with the Dutch Medical Research Involving Human Subjects Act. Adverse events are recorded in Castor; serious adverse events are reported to the central research team and included in annual safety reporting.

Recruitment began in March 2025; the last patient is expected to be included in January 2027, with data collection concluding by January 2029. Data are handled in accordance with the General Data Protection Regulation (European Union) 2016/679. Independent monitoring is performed by the Amsterdam UMC Clinical Monitoring Center, following the study’s monitoring plan (online supplemental appendix 3: monitoring plan). Baseline data are extracted from hospital records and questionnaires; follow-up data come from general practitioners and participant questionnaires. Source documents are securely stored at study sites, while follow-up data are centrally managed at Amsterdam UMC. All study data will be archived for 25 years. Deidentified data will be made available on reasonable request, in line with ethical approvals and data protection regulations. Further details on data management, storage and governance are available in online supplemental appendix 4: data management plan.

The results of this trial will be submitted for publication in peer-reviewed open access journals and presented at national and international conferences. In collaboration with the Dutch Brain Injury Association, findings will also be disseminated through patient networks and the association’s website to reach a broader public. This study will provide valuable insights and have direct implications for clinical practice. Results will be integrated into Dutch national guidelines, facilitating implementation in routine care.

## Discussion

The SAFEST-RCT evaluates the effectiveness of starting statin therapy after ischaemic stroke or TIA in frail older adults, a population typically excluded from trials.^8^ With no clear guidelines for this population,^32 33^ statin prescribing varies widely in this group. This trial aims to reduce such variability by providing clear clinical guidance.

Due to a lack of clear guidelines on statin discontinuation, management practices also vary for patients already on statins at the time of their event.^32 33^ Initially, we intended to study both initiation and discontinuation of statins within one RCT. However, ethical concerns prevented randomisation for discontinuation. As a result, we are now conducting ‘SAFEST—the prospective cohort study’ alongside the SAFEST-RCT. This observational study investigates continuing versus discontinuing statins in frail older adults already on statin therapy at the time of their ischaemic stroke or TIA. Data from this cohort will be used to emulate a trial estimating the effects of discontinuing.^57^

Our study has several strengths. Its randomised design minimises selection bias, and the multicentre inclusion of 22 academic and general hospitals across the Netherlands enhances representativeness of the national population. This includes individuals with a migration background, provided they are proficient in Dutch or supported by a proxy. The use of the validated CFS to assess frailty strengthens the study’s methodology.^35^ Given the international use of the CFS and the comparability of Dutch healthcare to other high-income countries, findings are likely generalisable beyond the Netherlands.^58^ To our knowledge, no other trials are currently investigating the effect of statins following an ischaemic stroke or TIA in frail older adults.

This study has several limitations. First, its open-label design may introduce bias, as patients’ awareness of their treatment allocation could influence the perceived benefits, adverse effects and responses to the patient-reported PROMIS-10 questionnaire, which is the primary outcome in this study. This design was chosen to minimise patient burden by avoiding placebo use and to prevent confusion in medication management, which is particularly important in frail older adults. The direction and magnitude of this potential bias are uncertain; participants’ beliefs about their treatment may affect questionnaire responses either positively or negatively. To minimise bias, outcome assessments are conducted by study team members who remain blinded to treatment allocation. Second, the exclusion of individuals unable to provide informed consent, such as those with severe cognitive impairment or disabling aphasia, may limit the generalisability of findings to the most severely affected populations. Third, the exclusion of patients unable to communicate in Dutch may introduce bias, as non-Dutch-speaking residents could have a different profile of stroke risk factors and comorbidities. To minimise this risk, patients with limited Dutch proficiency are allowed to participate with support from a proxy. Fourth, adherence to statin therapy will be assessed through pharmacy dispensing records, which reflect prescription collection but not actual medication intake. This approach was chosen to minimise participant burden in this frail population, in which more direct methods, such as blood tests, pill counts or adherence questionnaires, as described in the review of López-Pineda *et al*,^59^ were not feasible. Fifth, in the intervention group, the choice of statin type and dose is left to the treating neurologist according to routine care and the Dutch Stroke guideline, which may introduce some heterogeneity in treatment. Lastly, the study focuses solely on statin therapy and does not consider other lipid-lowering agents such as ezetimibe, bempedoic acid or proprotein convertase subtilisin/kexin type 9 inhibitors, which may be explored in future research.

In summary, the SAFEST-RCT and its complementary cohort study aim to fill a critical evidence gap in the management of statin therapy in frail older adults after ischaemic stroke or TIA, to provide essential guidance for clinical decision-making in this vulnerable and understudied population.

## Supplementary material

10.1136/bmjno-2025-001297 online supplemental file 1

10.1136/bmjno-2025-001297 online supplemental file 2

10.1136/bmjno-2025-001297 online supplemental file 3

10.1136/bmjno-2025-001297 online supplemental file 4
