# Supplementary material for: Rationale and design of ‘StAtins in Frail oldEr patients with ischemic Stroke or Transient ischemic attack–the Randomized Controlled Trial’ (SAFEST-RCT)
Source: BMJ Neurol Open. 2025 Oct 5;7(2):e001297. doi: 10.1136/bmjno-2025-001297 (PMC12506154; doi:10.1136/bmjno-2025-001297)
Supplement: online supplemental file 1 [file bmjno-7-2-s001.docx]

**Supplementary Appendix 1. Participating study sites and local principal investigators**

| **Study site** | **Principal investigator** |
| --- | --- |
| Amsterdam University Medical Center | Paul J. Nederkoorn |
| Catharina Hospital | Rob A. Gons |
| Elisabeth-TweeSteden Hospital | Ben P.W. Jansen |
| Elkerliek Hospital | Maaike Bos |
| FlevoHospital | Elizabeth Osei |
| Fransiscus Gasthuis en Vlietland | Kirsten R.I.S. Dorresteijn |
| Gelre Hospitals | Renske G. Wieberdink |
| Haaglanden Medical Center | Raoul Kloppenborg |
| Isala Clinics | Wilmar Jolink |
| Jeroen Bosch Hospital | Marian S.G. van Zagten |
| Leiden University Medical Center (LUMC) | Nyika D. Kruyt |
| Maasstad Hospital | Walid Moudrous |
| Maastricht University Medical Center+ (MUMC+) | Julie Staals |
| Frisius Medical Center | Frank G. van Rooij |
| Medisch Spectrum Twente | Renate M. Arntz |
| OLVG | Renske M. Van den Berg-Vos |
| Rijnstate Hospital, Arnhem | Sarah E. Vermeer |
| St. Antonius Hospital, Nieuwegein | Marjon van der Meulen |
| University Medical Center Groningen (UMCG) | Suzanne Persoon |
| University Medical Center Utrecht (UMCU) | H. Bart van der Worp |
| VieCuri Medical Center | Floris de Kleermaeker |
| Zuyderland Medical Center | Tobien Schreuder |
