# Supplementary material for: Rationale and design of ‘StAtins in Frail oldEr patients with ischemic Stroke or Transient ischemic attack–the Randomized Controlled Trial’ (SAFEST-RCT)
Source: BMJ Neurol Open. 2025 Oct 5;7(2):e001297. doi: 10.1136/bmjno-2025-001297 (PMC12506154; doi:10.1136/bmjno-2025-001297)
Supplement: online supplemental file 2 [file bmjno-7-2-s002.docx]

[Logo deelnemend ziekenhuis]

**Proefpersoneninformatie voor deelname
aan medisch-wetenschappelijk onderzoek**

StAtins in Frail oldEr patients with ischemic Stroke or Transient ischemic attack – the randomized controlled trial (SAFEST-RCT)

Statines bij oudere patiënten met verminderde veerkracht na een herseninfarct of TIA


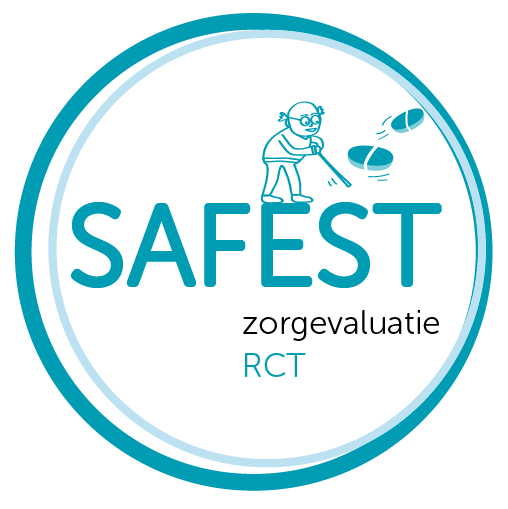


**Inleiding**

Geachte lezer,

Met deze informatiebrief willen we u vragen of u wilt meedoen aan medisch-wetenschappelijk onderzoek. Meedoen is vrijwillig. U krijgt deze brief omdat u 70 jaar of ouder bent, recent een herseninfarct of TIA heeft gehad, en daarvóor nog geen cholesterolverlagend medicijn (statine) gebruikte. U kunt meedoen aan het onderzoek als wij inschatten dat u verminderde veerkracht heeft. De veerkracht wordt bepaald voorafgaand aan het onderzoek.

U leest hier om wat voor onderzoek het gaat, wat het onderzoek voor u betekent en wat er van u verwacht wordt wanneer u besluit om deel te nemen.

**Stel uw vragen**

U kunt uw beslissing nemen met de informatie die u in deze informatiebrief vindt. Daarnaast raden we u aan om dit te doen:

- Stel vragen aan de onderzoeker die u deze informatie geeft.

- Praat met uw partner, familie of vrienden over dit onderzoek.

- Lees deze brief dan aandachtig door, en lees eventueel de informatie op [www.rijksoverheid.nl/mensenonderzoek](http://www.rijksoverheid.nl/mensenonderzoek).

Wilt u meedoen? Vul dan het formulier in de bijlage in.

1. **Algemene informatie**

Amsterdam UMC heeft dit onderzoek opgezet. Hieronder noemen we Amsterdam UMC steeds de ‘opdrachtgever’. Onderzoekers, dit kunnen ook artsen of onderzoeksverpleegkundigen zijn, voeren het onderzoek uit in 24 verschillende ziekenhuizen. Deelnemers aan een medisch-wetenschappelijk onderzoek worden vaak proefpersonen genoemd. Zowel patiënten als mensen die gezond zijn, kunnen proefpersoon zijn. Voor dit onderzoek zijn 600 proefpersonen nodig. De medisch-ethische toetsingscommissie van Amsterdam UMC heeft dit onderzoek goedgekeurd.

1. **Wat is het doel van het onderzoek?**

Statines worden gebruikt om de kans op (nieuwe) hart- en vaatziekten te verkleinen. Bij jongere patiënten is deze behandeling bewezen effectief en geven statines over het algemeen weinig ernstige bijwerkingen. Het is onbekend of dit net zo zeer geldt voor oudere patiënten met verminderde veerkracht die recent een herseninfarct of TIA hebben gehad.

Het doel van dit onderzoek is om het effect van een behandeling met een statine in deze groep patiënten te bestuderen. We willen onderzoeken wat de gevolgen zijn van het starten of niet starten met een statinebehandeling. We bestuderen hiervoor kwaliteit van leven en de overleving zonder nieuwe hart- en vaatziekten, tot 2 á 3 jaar na een herseninfarct of TIA.

1. **Hoe verloopt het onderzoek?**

*Hoelang duurt het onderzoek?*

Doet u mee met het onderzoek? Dan duurt dat in totaal tenminste twee jaar. Voor de mensen die in het begin van de studie starten met de studie, duurt het onderzoek drie jaar.

*Stap 1: bent u geschikt om mee te doen?*

We willen eerst weten of u geschikt bent om mee te doen. Daarvoor controleert de onderzoeker uw veerkracht met behulp van een checklist, de Clinical Frailty Scale (CFS). Met deze checklist wordt de mate van zelfstandigheid, functioneren en algehele gezondheid van een persoon ingeschat. Op basis van uw score op deze schaal kunnen we bepalen of u in aanmerking komt voor deelname aan het onderzoek.

*Stap 2: de behandeling*

Als u meedoet aan het onderzoek dan wordt u behandeld met de gebruikelijke medicijnen na een herseninfarct of TIA, met daarbij wel óf geen statine gedurende twee jaar, of in sommige gevallen drie jaar.

Voor dit onderzoek maken we 2 groepen:

- Groep 1. De mensen in deze groep krijgen een statine. Dit is het advies volgens de richtlijn “Herseninfarct en hersenbloeding”.
- Groep 2. De mensen in deze groep krijgen geen statine. Dit is het advies volgens de richtlijn “Cardiovasculair risicomanagement (CVRM)".

Loting bepaalt in welke groep u belandt.

*Stap 3: onderzoeken*

Tijdens dit onderzoek vult u vragenlijsten in en houdt u een kalender bij:

- Aan het begin van de studie, wanneer u in het ziekenhuis bent, vult u vier vragenlijsten in over uw kwaliteit van leven, geestelijk en lichamelijk functioneren. Dit zal maximaal 30 minuten duren.
- In de twee jaar daarna vult u op vijf momenten opnieuw vragenlijsten in, via de telefoon of de post. Dit zal drie keer 5 minuten duren, en twee keer 30 minuten.
- U houdt op een speciale kalender bij wanneer u valt of een eventuele nieuwe hart- en vaatziekte krijgt. U verstuurt deze kalender elke 3 maanden naar de onderzoekers via de post. Dit gaat via een antwoordnummer. Er zijn daardoor geen frankeerkosten aan verbonden.
- Indien u tot de eerste deelnemers behoort die aan het begin van de studie zijn gestart, kunnen we u vragen om nog een derde jaar mee te doen aan de studie, en 3 jaar na deelname nog één extra keer vragen over uw kwaliteit van leven en hart- en vaatziekten te beantwoorden. Dit gebeurt via de telefoon of via de post en dit duurt in totaal maximaal 10 minuten.

Verder gebeurt er tijdens het onderzoek het volgende:

- Aan het begin van de studie verzamelen wij informatie over uw leeftijd, geslacht, type herseninfarct of TIA, rookgedrag en alcoholgebruik, ziektegeschiedenis, lengte, gewicht, bloeddruk, en bloedwaarden (zoals de hoogte van het cholesterol, glucose, lever- en nierwaarden) uit uw medisch dossier.
- We bewaren gegevens over uw naam, en uw contactgegevens om contact met u op te nemen voor de controlemomenten
- Wij hebben twee of drie keer contact met uw huisarts of andere behandelend arts om bloedwaarden en bloeddruk op te vragen en vragen te stellen over eventuele hart- en vaatziekten.
- We nemen tweemaal contact op met uw apotheek om te vragen naar eventuele wijzigingen in uw medicatie. Het kan ook zijn dat we in plaats daarvan pas aan het einde van de studie informatie opvragen over uw medicijngebruik. Dat doen we dan bij de Stichting Farmaceutische Kengetallen, een organisatie die gegevens verzamelt over medicijngebruik in Nederland, en is eenmalig.

*Wat is er anders dan bij gewone zorg?*

Normaal gesproken hangt de beslissing om wel of geen statine te gebruiken af van wat uw arts kiest. Sommige artsen volgen de richtlijn "Herseninfarct en Hersenbloeding" en schrijven meestal een statine voor. Andere artsen volgen de richtlijn "CVRM" en schrijven meestal geen statine voor.

Als u meedoet aan dit onderzoek, wordt de keuze om wel of geen statine te gebruiken bepaald door loting (toeval), in plaats van door uw arts. Wat verder anders is, is dat u tijdens de twee jaar dat u meedoet, een aantal keer contact heeft met een arts-onderzoeker om vragenlijsten in te vullen.

1. **Welke afspraken maken we met u?**

We willen graag dat het onderzoek goed verloopt. Daarom maken we de volgende afspraken met u:

- Als u in de groep belandt die een statine gaat gebruiken, neemt u het medicijn op de manier die de arts u heeft uitgelegd.
- U doet tijdens dit onderzoek niet mee aan een ander medisch-wetenschappelijk onderzoek.
- U neemt contact op met de onderzoeker in de volgende situaties:
  - U wilt niet meer meedoen met het onderzoek.
  - Uw telefoonnummer, adres of e-mailadres verandert.

1. **Van welke bijwerkingen, nadelige effecten of ongemakken kunt u last krijgen?**

De volgende bijwerkingen komen soms voor tijdens het slikken van een statine:

- - Spierpijnklachten
  - Maagdarmklachten
  - Verhoogde suikers in het bloed
  - Hoofdpijn

Meer informatie over bijwerkingen van de statine staat in de bijsluiter.

1. **Wat betekent deelname voor u?**

U heeft zelf geen voordeel van meedoen aan dit onderzoek. Uw deelname helpt ons om betere aanbevelingen te doen voor de behandeling van oudere patiënten met verminderde veerkracht die een herseninfarct of TIA hebben gehad.

*Mogelijke voordelen van het gebruiken van een statine:*

Een statine zou de kans op een nieuwe hart en vaatziekte kunnen verlagen, maar zeker is dat niet.

*Mogelijke nadelen van het gebruiken van een statine:*

Een statine zou kunnen zorgen voor bijwerkingen of veranderde werking van andere medicijnen die u gebruikt.

*Mogelijke voordelen van het meedoen aan de studie:*

Tijdens de studie heeft u frequent contact met (arts-)onderzoekers, wat een gevoel van veiligheid en ondersteuning kan bieden in uw zorgtraject. Verder helpt uw deelname ons om betere aanbevelingen te doen voor de behandeling van oudere patiënten met verminderde veerkracht die een herseninfarct of TIA hebben gehad.

*Mogelijke nadelen van het meedoen aan de studie:*

Deelname aan de studie vereist tijd voor het invullen van vragenlijsten, het bijhouden van een kalender en het contact met (arts-)onderzoekers, wat als belastend kan worden ervaren.

1. **Als u niet mee wilt doen of wilt stoppen met het onderzoek.**

Deelname aan het onderzoek is geheel vrijwillig. Wilt u niet meedoen, dan krijgt u de gewone behandeling. Uw arts besluit dan (samen met u) of u start met een statine.

1. **Wanneer stopt het onderzoek?**

De onderzoeker laat het u weten als er nieuwe informatie over het onderzoek komt die belangrijk voor u is. De onderzoeker vraagt u daarna of u blijft meedoen.

*In deze situaties stopt voor u het onderzoek:*

- Alle onderzoeken volgens het schema zijn voorbij.
- Het einde van het hele onderzoek is bereikt.
- U wilt zelf stoppen met het onderzoek. Dat mag op ieder moment. Meld dit dan meteen bij de onderzoeker. U hoeft er niet bij te vertellen waarom u stopt. De gegevens die tot dat moment zijn verzameld, worden wel gebruikt voor het onderzoek.
- De onderzoeker vindt het beter voor u om te stoppen.
- Een van de volgende instanties besluit dat het onderzoek moet stoppen:
  - Amsterdam UMC
  - de overheid, of
  - de medisch-ethische commissie die het onderzoek beoordeelt.

1. **Wat gebeurt er na het onderzoek?**

*Kunt u de medicijnen blijven gebruiken?*

Als u tijdens dit onderzoek een statine heeft gebruikt, mag u dit na het onderzoek blijven gebruiken. Als u tijdens dit onderzoek geen statine heeft gebruikt, maar u wilt daar na het onderzoek wel mee starten, mag dat ook, in overleg met de behandelend (huis)arts.

*Krijgt u de resultaten van het onderzoek?*

Ongeveer een jaar nadat het onderzoek is afgerond laat de onderzoeker u weten wat de belangrijkste uitkomsten zijn van het onderzoek.

1. **Wat doen we met uw gegevens?**

*Doet u mee met het onderzoek?*

Dan geeft u ook toestemming om uw gegevens te verzamelen, gebruiken en bewaren.

*Waarom verzamelen, gebruiken en bewaren we uw gegevens?*

We verzamelen, gebruiken en bewaren uw gegevens om de vragen van dit onderzoek te kunnen beantwoorden. De resultaten van het onderzoek willen we publiceren in een medisch tijdschrift.

*Hoe beschermen we uw privacy?*

Om uw privacy te beschermen zullen wij uw persoonlijke gegevens gecodeerd bewaren. Dit betekent dat we de gegevens een code geven. De sleutel van de code bewaren we op een beveiligde plek in het ziekenhuis. Als we uw gegevens verwerken gebruiken we steeds alleen die code. Ook in rapporten en publicaties over het onderzoek kan niemand terughalen dat het over u ging. Uw contactgegevens worden wel met de onderzoeksgroep gedeeld, zodat er contact met u kan worden opgenomen, voor het afnemen van de vragenlijsten.

*Wie kunnen uw gegevens zien?*

Sommige personen kunnen wel uw naam en andere persoonlijke gegevens zonder code inzien. Dit kunnen gegevens zijn die speciaal voor dit onderzoek zijn verzameld, maar ook gegevens uit uw medisch dossier. Deze personen kunnen bij uw gegevens komen:

Het lokale onderzoeksteam, dat betrokken is bij de dataverzameling aan het begin van de studie.

Een lid van het onderzoeksteam uit het Amsterdam UMC, die de verdere dataverzameling doet, en die telefonisch of per post contact met u opneemt voor de vragenlijsten en kalender.

Leden van de commissie die de veiligheid van het onderzoek in de gaten houdt.

Een controleur die door de onderzoeker is ingehuurd.

Deze personen houden uw gegevens geheim. Voor inzage door deze personen vragen wij u toestemming te geven. De Inspectie Gezondheidszorg en Jeugd kan zonder uw toestemming uw gegevens inzien.

*Hoe lang bewaren we uw gegevens?*

Uw gegevens worden 25 jaar bewaard. Gegevens die bij de start van het onderzoek zijn verzameld, blijven in het ziekenhuis waar u aan het onderzoek bent begonnen. Gegevens die tijdens de verdere controle worden verzameld, worden in Amsterdam UMC bewaard.

*Mogen we uw gegevens gebruiken voor ander onderzoek?*

De gegevens die 25 jaar bewaard worden in deze twee ziekenhuizen kunnen na afloop van dit onderzoek ook nog van belang zijn voor ander wetenschappelijk onderzoek op het gebied van ouderen met verminderde veerkracht en een herseninfarct of TIA. Op het toestemmingformulier kunt u aangeven of u het goed vindt dat uw gegevens worden gebruikt voor ander onderzoek. Geeft u hiervoor geen toestemming? Dan kunt u nog steeds meedoen met dit onderzoek.

*Kunt u uw toestemming voor het gebruik van uw gegevens weer intrekken?*

U kunt uw toestemming voor het gebruik van uw gegevens op ieder moment intrekken. Dit geldt voor het gebruik in dit onderzoek en voor het gebruik in ander onderzoek. Maar, trekt u uw toestemming in, en hebben de onderzoekers al gegevens verzameld voor dit onderzoek? Dan mogen zij deze gegevens nog wel gebruiken.

*Mogen wij u na dit onderzoek opnieuw benaderen voor een vervolgonderzoek?*

Wanneer dit onderzoek is afgelopen, doen we misschien een vervolgonderzoek. We willen u dan benaderen met de vraag of u weer deel wilt nemen. Op het toestemmingsformulier kunt u aangeven of u ons daar toestemming voor geeft. Geeft u hiervoor geen toestemming? Dan kunt u nog steeds meedoen met dit onderzoek.

*Wilt u meer weten over uw privacy?*

- Wilt u meer weten over uw rechten bij de verwerking van persoonsgegevens? Kijk dan op [www.autoriteitpersoonsgegevens.nl](http://www.autoriteitpersoonsgegevens.nl).
- Heeft u vragen over uw rechten? Of heeft u een klacht over de verwerking van uw persoonsgegevens? Neem dan contact op met degene die verantwoordelijk is voor de verwerking van uw persoonsgegevens. Voor uw onderzoek is dat:
  - [Naam instelling] Zie bijlage A voor contactgegevens.
- Als u klachten heeft over de verwerking van uw persoonsgegevens, raden we u aan om deze eerst te bespreken met het onderzoeksteam. Er kan contact worden opgenomen met de Functionaris Gegevensbescherming van Amsterdam UMC via privacy@amsterdamumc.nl. Of u dient een klacht in bij de Autoriteit Persoonsgegevens.

1. **Krijgt u een vergoeding voor meedoen?**

U krijgt geen vergoeding voor het meedoen aan dit onderzoek.

Aangezien u voor het onderzoek niet extra naar het ziekenhuis hoeft te komen, is er ook geen vergoeding voor aanvullende reiskosten.

1. **Bent u verzekerd tijdens het onderzoek?**

U bent niet extra verzekerd voor dit onderzoek. Want als u meedoet aan het onderzoek, heeft u dezelfde risico’s als bij de gewone behandeling van uw herseninfarct of TIA. Daarom hoeft de opdrachtgever van de medisch ethische commissie van het Amsterdam UMC geen extra verzekering af te sluiten.

1. **We informeren uw huisarts**

De onderzoeker stuurt uw huisarts een brief om te laten weten dat u meedoet aan het onderzoek.

1. **Heeft u vragen?**

over het onderzoek kunt u stellen aan het onderzoeksteam. Wilt u advies van iemand die er geen belang bij heeft? Ga dan naar de onafhankelijk deskundige. Zij weet veel over het onderzoek, maar werkt niet mee aan dit onderzoek.

1. **Heeft u een klacht?**

Mocht u een klacht hebben, bespreek die dan met de onderzoeker of de arts die u behandelt.

Wilt u dit liever niet? Ga dan naar de klachtencommissie van uw ziekenhuis. In bijlage A staat waar u die kunt vinden.

1. **Hoe geeft u toestemming voor het onderzoek?**

U kunt eerst rustig nadenken over dit onderzoek. Daarna vertelt u de onderzoeker of u de informatie begrijpt en of u wel of niet wilt meedoen. Wilt u meedoen? Dan vult u het toestemmingsformulier in dat u bij deze informatiebrief vindt. U en de onderzoeker krijgen allebei een getekende versie van deze toestemmingsverklaring.

Dank voor uw aandacht.

**Contactgegevens:**

*Lokale hoofdonderzoeker SAFEST – RCT binnen [naam instelling]*

Naam: [naam lokale hoofdonderzoeker]

Telefoonnummer: [telefoonnummer lokale hoofdonderzoeker]

E-mail: [mail adres lokale hoofdonderzoeker]

*Hoofdonderzoeker SAFEST - RCT*

Naam: Prof. dr. R. M. van den Berg-Vos, neuroloog OLVG en Amsterdam UMC

Telefoonnummer: 020-5108780

E-mail: r.vandenberg-vos@olvg.nl

*Onafhankelijk deskundige SAFEST - RCT*

Naam: Dr. Marieke C. Visser, neuroloog Amsterdam UMC

E-mail: [mc.visser@amsterdamumc.nl](mailto:mc.visser@amsterdamumc.nl)

**Bijlage A: contactgegevens voor [naam deelnemend centrum]**

**Onderzoeker**

[Hoofdonderzoeker van centrum: naam, contactgegevens en bereikbaarheid]

< *indien van toepassing*>

[ Onderzoeksverpleegkundige/ onderzoeksarts/ verpleegkundig specialist: naam, contactgegevens en bereikbaarheid]

**Klachten**:

Patiëntenservice Zorgsupport locatie AMC bereikbaar PAZOAMC@amsterdamumc.nl

**Verwerkingsverantwoordelijke**:
Raad van Bestuur van Amsterdam UMC

**Functionaris voor de Gegevensbescherming van de instelling**:

[Naam en contactgegevens van de Functionaris voor de Gegevensbescherming]

**Functionaris voor de Gegevensbescherming van de verrichter:**

Amsterdam UMC via privacy@amsterdamumc.nl.

**Voor meer informatie over uw rechten**:

[Contactgegevens [inclusief website] van de verantwoordelijke(n) voor de verwerking van persoonsgegevens]:

<*indien van toepassing aan te vullen met bijvoorbeeld coördinerend onderzoeker en/of een alarmnummer/24-uur bereikbaarheid*>

**Toestemmingsformulier proefpersoon**

Behorende bij SAFEST-RCT

- Ik heb de informatiebrief gelezen. Ook kon ik vragen stellen. Mijn vragen zijn voldoende beantwoord. Ik had genoeg tijd om te beslissen of ik meedoe.
- Ik weet dat meedoen vrijwillig is. Ook weet ik dat ik op ieder moment kan beslissen om toch niet mee te doen of te stoppen met het onderzoek. Daarvoor hoef ik geen reden te geven.
- Ik geef toestemming om mijn huisarts te laten weten dat ik meedoe aan dit onderzoek.
- Ik geef toestemming aan mijn behandelend arts om mijn behandeling met de onderzoekers te bespreken.
- Ik geef toestemming voor het verzamelen en gebruiken van mijn gegevens op de manier en voor de doelen die in de informatiebrief staan, inclusief:
  - het overnemen van basisgegevens uit mijn medisch dossier
  - het opvragen van gegevens bij mijn huisarts of behandelend arts
  - het opvragen van gegevens betreffende mijn medicijngebruik bij mijn apotheek of bij de Stichting Farmaceutische Kengetallen, een organisatie die gegevens verzamelt over medicijngebruik in Nederland

De onderzoekers doen dit alleen om de onderzoeksvraag van dit onderzoek te beantwoorden.

- Ik weet dat voor de controle van het onderzoek sommige mensen al mijn gegevens kunnen inzien. Die mensen staan in deze informatiebrief. Ik geef deze mensen toestemming om mijn gegevens in te zien voor deze controle.
- Ik geef toestemming om mijn gegevens nog 25 jaar na dit onderzoek te bewaren binnen het Amsterdam UMC en het ziekenhuis waar ik het onderzoek ben gestart.
- Ik wil meedoen aan dit onderzoek.

Wilt u hieronder ja of nee aankruisen op de aanvullende vragen. Ook als u hier nee aankruist, kunt u meedoen met dit onderzoek.:

- Ik geef toestemming om mijn gegevens te gebruiken voor ander onderzoek, zoals in de informatiebrief staat vermeld

O ja O nee

- Ik geef toestemming om mij na dit onderzoek opnieuw te benaderen voor vervolgonderzoek

O ja O nee

Naam proefpersoon:

Handtekening: Datum : __ / __ / __

-----------------------------------------------------------------------------------------------------------------

Ik verklaar dat ik deze deelnemer volledig heb geïnformeerd over het genoemde onderzoek.

Als er tijdens het onderzoek informatie bekend wordt die de toestemming van de deelnemer zou kunnen beïnvloeden, dan breng ik hem/haar daarvan tijdig op de hoogte.

Naam onderzoeker:

Handtekening: Datum: __ / __ / __

-----------------------------------------------------------------------------------------------------------------

*De deelnemer krijgt een volledige informatiebrief mee, samen met een kopie van het getekende toestemmingsformulier.*
