# Supplementary material for: Rationale and design of ‘StAtins in Frail oldEr patients with ischemic Stroke or Transient ischemic attack–the Randomized Controlled Trial’ (SAFEST-RCT)
Source: BMJ Neurol Open. 2025 Oct 5;7(2):e001297. doi: 10.1136/bmjno-2025-001297 (PMC12506154; doi:10.1136/bmjno-2025-001297)
Supplement: online supplemental file 3 [file bmjno-7-2-s003.pdf]

REGULAR MONITORING PLAN
Investigator initiated studies

This form is developed by the Amsterdam UMC - Clinical Monitoring Center and intended for the use of employees of the Clinical Monitoring Center only. The document is confidential; nothing from this document may be copied, shared or distributed without written authorization of the Amsterdam UMC - Clinical Monitoring Center.

**Study** *(title and acronym)*	StAtins in Frail oldEr patients with ischemic Stroke or Transient ischemic attack – the randomized controlled trial<br>SAFEST-RCT
**Dossier number**	2024-517343-31-00
**Review committee number**	-
**Sponsor**	Amsterdam UMC
**Department**	Neurology
**Head of department**	Prof. D. van de Beek, MD, PhD
**Coordinating principal investigator**	Prof. R.M. van den Berg-Vos, MD, PhD
**Coordinating investigator / study coordinator**	Prof. N. van der Velde, MD, PhD/<br>S. Prins, MD, PhD candidate
**Type of study**	Medicinal product (phase IV, low-intervention trial)*<br><br>* The study is part of the program Zorgevaluatie en Gepast Gebruik (ZE&GG), which means that the Nederlandse Vereniging voor Neurologie (NVN) and the Nederlandse Vereniging voor Klinische Geriatrie (NVKG)determined that the intervention in this study is existing care.
**Risk classification**	Negligible
**Monitoring plan version and date**	Version 1.0, dated 20MAR2025

Table of content

1. Purpose of the monitoring plan 3
2. Clinical Monitoring Center service 3
3. Estimated enrolment and participating sites 3
4. Risk classification and risk management 4
5. General monitoring procedures 4
   5.1. Study-specific monitoring visit schedule/ monitoring frequency 5
6. Monitoring responsibilities and tasks 6
   6.1. Enrolment progress 6
   6.2. Trial master file (TMF)/ investigator site file (ISF) 6
   6.3. Informed consent 6
   6.4. Informed consent (IC) process and execution 6
   6.5. Inclusion and exclusion criteria 6
   6.6. Source data review and source data verification 7
   6.7. Safety reporting 8
   6.8. Investigational medicinal product under investigation 9
   6.9. Equipment and facilities 9
   6.10. Laboratory 9
   6.11. Pharmacy 9
   6.12. Privacy legislation 9
   6.13. Data validity/ integrity 9
7. Closing and reporting 9
   7.1. Resolution of reported monitoring issues 10
   7.2. Escalation of unresolved monitoring issues 10
8. Approval form 11
Appendix I: Types of monitoring 12
Appendix II: Participating sites 14

1. Purpose of the monitoring plan

The primary objective of this monitoring plan is to support the conduct of investigator initiated studies. This monitoring plan facilitates compliance with the Human Research Act (WMO), Good Clinical Practice (ICH-GCP) guidelines, and/or ISO14155, which require clinical research associates (CRA) to verify that:

(a) The rights and well-being of human subjects are protected.
(b) Recorded study data is accurate, complete, and verifiable with the source documents.
(c) The conduct of the study is compliant with the currently approved study protocol and with the applicable laws and regulatory requirements, e.g. WMO, ICH-GCP and/or ISO14155.

This monitoring plan is used by the CRA while monitoring investigational sites that participate in this study. This document describes the key monitoring activities and specifies the data to be verified over the course of the clinical study. The monitoring activities are in principle based on the additional risk of this study, compared to standard treatment and are described in the current NFU-guideline '*Kwaliteitsborging van Mensgebonden Onderzoek*'.

The study-specific monitoring plan is updated and revised as needed. The most recent version of the monitoring plan will take precedence over any previous version(s).

2. Clinical Monitoring Center service

The Clinical Monitoring Center (CMC) supports clinical researches in order to comply with applicable laws and regulatory requirements, e.g. WMO, ICH-GCP and/or ISO14155.

As per Amsterdam UMC monitoring policy the monitoring within Amsterdam UMC, location AMC and VUmc, is free of charge. With regards to the monitoring costs for other participating sites, all parties agree on the costs as specified in the CMC quotation.

The CMC CRAs are independent from the study and the study team and have received at minimum an internal training specifically designed for their responsibilities and corresponding tasks. All CMC CRAs are WMO/ ICH-GCP certified and are trained in the principles of ISO14155. The coordinating principal investigator (PI) is responsible for providing specific therapeutic area and/or study protocol training, as well as training in the electronic case report form (eCRF), in case another system than Castor EDC is used. Training to the CRA can be provided by means of attending a site initiation visit, during the monitoring intake visit and/or by means of training by reading.

3. Estimated enrolment and participating sites

The CRA verifies, documents and reports the enrolment rate and the percentage of drop-outs (withdrawals and subjects lost to follow-up).

Estimated enrolment, according to applicable approved study protocol:

- Total number of study subjects to be enrolled: 612
- Inclusion duration: 2 years
- Expected inclusion rate: 25 study subjects per month

- Ethics committee approval date: 10DEC2024

- First study subject enrolled: MAR2025
- Study participation duration for study subject: 24 months (possibility 36 months)
- Last study subject enrolled: JAN2027
- Last study subject last visit: JAN2029 (2030)

- Final database lock: APR2029

*In the case the actual end of study date exceeds the estimated end of study date (i.e. last study subject last visit) specified in this study-specific monitoring plan, monitoring visits will be performed as outlined in paragraph 5.1 from this date until the end of study.

Details related to the sites participating in the study, the amount of study subjects planned to be enrolled per participating site, name and role of the contact person(s) and contact details are specified in appendix II: Participating sites.

4. Risk classification and risk management

The coordinating PI and/or delegate should identify the (potential) risks related to the study subject's safety and well-being, associated with the type and focus of the study protocol. This risk assessment results in a potential risk classification, which is described in the study protocol and is assessed by the applicable regulatory authorities.

In addition to the risks described in the study protocol, (potential) risks with regard to additional factors related to the study subject's rights, the quality and/or integrity of the collected study data and study management are discussed with the coordinating PI and/or delegate.

For all identified risks, discussed with the CRA during the monitoring intake visit, risk mitigation actions are pre-determined.

For this study protocol (potential) risks of special interest and the corresponding risk mitigation actions include the following:

**Study-specific risk**	**Risk mitigation**	**Responsible person** *<e.g. CRA, local study staff, principal investigator>*
Number of drop-outs during the study	Close monitoring of the responses during the follow up	Sponsor team (study coordinator)
Unblinding of the study coordinator	Study coordinator not to be involved in inclusion procedures and data collection	Sponsor team (study coordinator)

The (potential) risks related to a study protocol should be monitored and reviewed during the course of a study. During the course of the study (potential) risks are revised as necessary, e.g. in case new risks are identified, unanticipated risks emerge and/or anticipated risks do not occur. The identified risks can change during the course of the study as a result of any of the following (this list is not exhaustive):

- monitoring findings;
- audit or inspection findings;
- safety review and/or advice from the data safety monitoring board (DSMB);
- study protocol amendments;
- change in study staff, facilities or external service providers.

5. General monitoring procedures

By means of monitoring, the CRA verifies that a clinical study is conducted according to the applicable laws and regulations, is documented properly and that all documentation is maintained adequately. The monitoring process consists of several types of monitoring visits. Monitoring visits are conducted by the CRA on a regular basis, following site initiation until last study subject last visit and finalisation of study data entry in the (e)CRF. The different types of monitoring are specified in detail in appendix I: Types of monitoring.

For this study the following monitoring visits are agreed upon:

- **Site initiation visit(s)**
  The responsible coordinating PI or delegate of the sponsor site arranges and performs the site initiation visit(s), either in person or by means of providing all participating sites with the necessary study documentation, in combination with a web-based training and/or teleconference.

- **On-site monitoring visit(s)**
  Several on-site monitoring visits are scheduled and performed by the CRA allocated to the study, based on the current NFU-guideline '*Kwaliteitsborging van Mensgebonden Onderzoek*' (see section 5.1). For a specification of the monitoring activities that are performed during the visits, see section 6 of this monitoring plan.

- **Remote monitoring**
  Remote monitoring is utilised by the CRA as a method of maintaining oversight of a study or a participating site, in between the routine on-site monitoring visits. This includes the request for regular status updates from the coordinating PI or delegate on e.g. study subject enrolment and study protocol amendments, was well as regular communication with the participating site(s) on operational issues such as changes in study staff and the resolution of monitoring issues.

  In addition, for this study, remote monitoring for participating sites during the follow up includes central review of the eCRF and remote site monitoring Follow up data will be collected by the study team of the Amsterdam UMC. . Data to be verified consist of SAFEST calendar, study related questionnaires as specified in section 6.6.1 and documentation of contact with the general practitioner. .

- **Remote close out(s)**
  A remote close out is applicable to all participating sites, including the sponsor site. A customised remote site close out checklist is forwarded to the site to be closed by the CRA, for completion. The PI or delegate of the site to be closed returns a copy the completed checklist to the CRA, signed and dated by the local PI to confirm the study is adequately closed.

5.1. Study-specific monitoring visit schedule/ monitoring frequency

Amsterdam UMC

**Visit no.**	**Selected Sites**	**Planning***
Site initiation visit	Amsterdam UMC	Before enrolment of the first subject, but after approval is obtained from the applicable regulatory authorities and the local board of directors.
First on-site monitoring visit	Amsterdam UMC, location AMC	After enrolment of 3 subjects at site.
Second on-site monitoring visit	Amsterdam UMC, location AMC	After enrolment of 15-20 subjects at site.
Third on-site monitoring visit	Amsterdam UMC, location AMC	After last subject last visit.
Remote close out	Amsterdam UMC, location AMC	After last subject last visit.

*The monitoring visit schedule may be amended based on changes in the enrolment period, the inclusion rate, quality issues and/or site performance. Amendments in the monitoring visit schedule are discussed with and approved by the coordinating PI in writing.

Participating sites

**Visit no.**	**Selected Sites**	**Planning***
Site initiation visit	Participating sites	Before enrolment of the first subject, but after approval is obtained from the applicable regulatory authorities and the local board of directors.
First on-site monitoring visit	Participating sites	After enrolment of 5 subjects at site.

Visit no.	Selected Sites	Planning*
Remote visit	Participating sites	After enrolment of 15-20 subjects at site.
Remote visit	Participating sites	After last subject last visit.
Remote close out	Participating sites	After last study subject last visit.

6. Monitoring responsibilities and tasks

The specific monitoring tasks that apply to this study are outlined in the sections below.

6.1. Enrolment progress

The CRA verifies:

- the inclusion rate, if applicable the number of screen failures and the drop-out rate;
- if the reason for study withdrawal is adequately documented, if applicable;

6.2. Trial master file (TMF)/ investigator site file (ISF)

The CRA verifies the completeness of the TMF/ISF or ISF, based on CMC internal procedures; only specific essential documents of special interest are verified on presence and completeness by the CRA. Essential documents are maintained and filed by the PI or delegate of the sponsor site or participating site as specified in ICH-GCP section 8 and/or ISO 14155 annex E, as applicable.

6.3. Informed consent

6.3.1. Patient information form (PIF) and informed consent form (ICF)

The CRA verifies if:

- the approved version of the PIF/ICF is used;
- the PIF contains the proper investigational site-specific details;
- consent for access to medical records for the CRA, auditors and authorities (IGJ, EMA, FDA) is included in the PIF/ICF.

6.3.2. Informed consent form presence

The CRA verifies for at least 10% of the randomised study subjects if a signed and dated ICF is present.

6.4. Informed consent (IC) process and execution

The CRA verifies for an at random selection of at least 10% of the randomised study subjects if:

- the approved version of the ICF is used;
- the PI or delegate signed and dated the ICF at the same time as the study subject or the subject's representative;
- the ICF is personally signed and dated by the study staff member who informed the study subject or subject's representative about the study and performed the IC process;
- the ICF is personally signed and dated by a witness, if applicable;
- the ICF is signed and dated by the applicable parties prior to any study related procedure;
- exceptions in the IC process are documented in the study protocol and approved by the applicable regulatory authorities (e.g. deferred consent);
- the study subject's participation in the study is documented in the study subject's medical record;
- the IC process is documented in the study subject's medical record;
- the signed and dated original ICF is filed in the investigator subject site file;
- an amended version of the PIF/ICF is signed in a timely fashion by all study subjects who are still active in the study at the time of implementation of the amended version of the PIF /ICF, if applicable (re-consent).

6.5. Inclusion and exclusion criteria

The CRA verifies for an at random selection of at least 10% of the randomised study subjects if:

- the study subject fulfils all inclusion criteria and none of the exclusion criteria, according to the applicable approved study protocol;

- the PI or delegate confirmed the assessment of the inclusion and exclusion criteria in the medical record or (e)CRF.

6.6. Source data review and source data verification

6.6.1. Source documents

The CRA reviews the source documentation used and verifies if the source documentation is adequate and complete, in order to be able to verify study protocol compliance and critical processes are documented, verify the accuracy of the study data collected in the (e)CRF and to ascertain PI involvement and appropriate delegation of tasks and responsibilities of study staff members.

Source documents include, but are not limited to:

- electronic health records (EHR)
- other type of medical records (e.g. on paper);
- study related paper work sheets;
- study related questionnaires: EQ-5D-5L, T-MOCA, PROMIS-10, TOPICS-MDS, SAFEST calendars
- study related diaries;
- investigational (medicinal) product: local pharmacy contacts regarding medication dispensed

Print-outs of electronic data are not regarded as original source documents, unless they are signed and dated by a delegated study staff member. If study data is first recorded on paper and then transferred to an electronic database (e.g. electronic health record, questionnaire website), the paper copy is considered the original source document. In case of imaging procedures (e.g. CT, X-ray) the CRA reviews the associated report, not the actual images. The imaging reports must be kept by the site as source data.

The CRA verifies if source documents are accurate, complete, kept up to date and maintained.

6.6.2. Source data review (SDR)

The CRA performs source data review for an at random selection of at least 10% of the randomised study subjects.

The CRA verifies if:

- study protocol-specific procedures are performed according to the applicable study protocol;
- no forbidden concomitant medication is used or forbidden interventions are performed throughout the study, if applicable;
- study visits are performed within the study protocol specified visit windows, if applicable;
- medical decisions are documented by a physician, e.g. assessment of (S)AEs, subject enrolment/ withdrawal, physical examination, significance/ clinical relevance of laboratory test results, imaging reports.

6.6.3. Protocol deviations/ serious breaches

A protocol deviation/ serious breach is a failure to conduct all aspects of the study as described in the approved study protocol, the WMO, ICH-GCP and/or ISO14155, as applicable. The PI or delegate of the site where the protocol deviation/ serious breach occurred is responsible for documenting protocol deviation/ serious breach, reporting protocol deviation/ serious breach to the sponsor site and maintaining an overview of all protocol deviations and/or serious breaches that occurred at that site.

For multicentre studies, the coordinating PI approves or acknowledges all protocol deviations and/or serious breaches received from the participating sites and maintains an overall overview of all protocol deviations/ serious breaches that occurred throughout the study.

The CRA verifies:

- that protocol deviations/ serious breaches are documented correctly;
- whether significant protocol deviations and serious breaches are reported by the sponsor site to the regulatory authorities, if applicable.

6.6.4. Study protocol and study procedure training

The CRA verifies if:

- the responsibilities/ tasks for each study staff member are clearly defined and specified on the site signature and delegation log;
- each study staff member is trained on study protocol-specific procedures, as delegated by the local PI;
- training in the study protocol and applicable study procedures is documented (e.g. randomisation, unblinding, administration of I(M)P, study protocol-specific assessments);
- instructions for executing study protocol-specific procedures are present, if not applied in the routine clinical practice.

6.6.5. Source data verification (SDV)

For all study subjects selected for source data review (section 6.6.2.), the CRA verifies whether the study data entered in the (e)CRF is accurately reported and consistent with the source documents. Please note that the population selected for SDR/SDV might differ from the population selected for the verification of the inclusion and exclusion criteria.

The study data to verify as determined in consultation with the coordinating PI or delegate (a predefined list of variables, including the primary endpoint) are:

- general demographic data (such as age, gender) at baseline;
- inclusion and exclusion criteria;
- medical history and current medical conditions;
- randomisation result versus received treatment;
- primary endpoint: to evaluate the effect of initiating statin therapy versus no statin therapy on Major Adverse Cardiovascular Events (MACE)-free survival and health-related quality of life (HRQoL) over a two-year follow-up in frail individuals aged 70 and above with a recent ischemic stroke or TIA;
- concomitant medication;
- laboratory test result (at baseline)
- documentation of AEs/ SAEs/ SUSARs;
- documentation of Major Adverse Cardiovascular Events (MACE)
- Vital signs (length, height, systolic & diastolic blood pressure

The CRA documents which study subject data is verified, e.g. in the eCRF or on the paper CRF pages.

In case of excessive data entry errors in (e)CRF, the planned amount study subjects to verify might be increased for that specific site, in consultation with the coordinating PI. In this case, the monitoring plan is updated accordingly.

6.7. Safety reporting

SAEs/ SUSARs are collected, recorded and reported throughout the study period, defined as informed consent signature to study end. .

The CRA verifies:

- whether the sponsor has a detailed record of all AEs or laboratory anomalies that are critical to safety evaluations which are reported to him/her by the local investigator of the sponsor site and the local investigators of the participating sites (CTR: Article 41(3)).
- whether all SUSARs / unexpected events / urgent safety measures and at least 50% of the SAEs are recorded accurately in the (e)CRF, as per study protocol;
- whether the sponsor has notified all concerned local investigators and involved parties/participating local principal investigators of findings that could adversely affect the safety of the subjects.
- whether all SUSARs / unexpected events / urgent safety measures and at least 50% of the SAEs verified, are reported to the sponsor site within the timelines as per study protocol and applicable laws and/or regulations;
- whether at least 50% of the SAEs from participating sites are reported by the sponsor site in the Annual safety report (ASR) to the applicable regulatory authorities within the timelines, as per study protocol and applicable laws and/or regulations.

*Please note that SAEs which do not require immediate reporting are included cumulatively in the Annual Safety Report by the sponsor and submitted in CTIS.

If more than 3 SAEs are reported to the applicable regulatory authorities, but outside the required time lines, attention is paid to the SAE reporting process.

In case one unreported SAE is discovered, the CRA will additionally review, during that particular site monitoring visit, the source documents of 2 additional randomised study subjects not selected for SDR/SDV, for any unreported SAEs (if source documents are available). If, for these 2 additional study subjects verified, one or more unreported SAE are discovered, an additional site monitoring visit is planned in consultation with the coordinating PI, to verify all source documents of the study subjects randomised so far, for any unreported SAEs.

In case unreported SAEs are discovered, the coordinating PI or delegate is requested to report them as mandated by the study protocol and re-training of study staff is strongly recommended.

6.8. Investigational medicinal product under investigation

The CRA verifies if:

- the study staff is adequately trained in the randomisation procedure and obtaining the assigned investigational medicinal product;
- the date and if relevant time of randomisation is documented in the study subject's medical records
- the randomisation outcome is documented in the study subject's medical record;
- the prescription by the PI or delegate is in place;

6.9. Equipment and facilities

Not applicable

6.10. Laboratory

Not applicable

6.11. Pharmacy

Not applicable

6.12. Privacy legislation

For the sponsor site, the CRA verifies if all study documentation that include personal identifiers (e.g. name, date of birth, home address, medical records number) are kept in a separate binder, e.g. investigator subject site file (ISSF). This includes for example:

- signed and dated ICFs;
- the trial subject identification log;
- paper study subject diaries;
- paper study subject questionnaires;
- paper study subject declaration forms.

6.13. Data validity/ integrity

The CRA verifies if the eCRF used to collect and analyse study data is compliant with ICH-GCP/ ISO14155 and the Amsterdam UMC SOPs, as applicable.

7. Closing and reporting

After every monitoring visit, the CRA writes a site monitoring visit report. This report summarises the tasks performed by the CRA, contains a general description of the quality of the site and provides an overview of all monitoring issues/ findings/ discrepancies (further specified as monitoring issues) noted by the CRA. The monitoring issues are enlisted in an appendix and include a suggestion for a corrective action. The original signed and dated site monitoring visit report is sent per e-mail to the coordinating PI, with the head of department in copy.

For multicentre studies, a copy of the site monitoring visit report monitoring visit report additionally is sent in a separate e-mail to the site and/or location visited.

The recipient(s) should read the site monitoring visit report and file the report in the TMF/ISF or ISF, as applicable. In case of an audit or inspection, all site monitoring visit reports should be made available.

7.1. Resolution of reported monitoring issues

The study staff is responsible for adequate and timely follow-up of the monitoring issues enlisted in the appendix of the site monitoring visit report: 'monitoring issues and action tracker'. Monitoring issues are graded as either a high priority issue or a low priority issue;

- High priority issues should be resolved, or action to resolve the issue should at least be initiated, within 4 weeks upon receipt of the site monitoring visit report.
- Low priority issues should be resolved, or action to resolve the issue should at least be initiated, preferably within 12 weeks, but at least prior to the next on-site monitoring visit.

In case the assessment of a monitoring issue by the CRA is inconclusive, the monitoring issue is discussed with the head of department CMC or delegate.

When all high priority issues are resolved or action to resolve the monitoring issue is initiated, the completed and signed 'Monitoring Issues and Action Tracker' (appendix to the site monitoring visit report) is returned to the CRA (in PDF-format).

Follow-up of all monitoring issues enlisted in the signed 'monitoring issues and action tracker' is verified by the CRA upon receipt and during the next on-site monitoring visit, as applicable.

In case of unconfirmed, outstanding monitoring issues at the time of a remote site close out, the outstanding monitoring issues are included as appendix to the remote close out checklist. The PI's confirmation for the resolution of the outstanding monitoring issues is requested. The completed and signed appendix is returned to the CRA (in PDF-format) and filed in the TMF/ISF and ISF, as applicable.

7.2. Escalation of unresolved monitoring issues

In the case that high priority issues are not resolved or no action to resolve the monitoring issues is initiated within 4 weeks after receipt of the site monitoring visit report, a kind reminder is sent to the responsible PI to return the completed and signed 'monitoring issues and action tracker' to the CRA.

If no action is taken in response to the reminder within the 2 subsequent weeks, a second reminder is sent to the responsible PI by:

- the head of department CMC, in case the participating site is the sponsor site, or
- the coordinating PI, in case of a participating sites other than the sponsor site.

In case no response is obtained to the second reminder within another 2 weeks, the 'monitoring issues and action tracker' is forwarded to the Amsterdam UMC QA manager and applicable measures are initiated. When deemed required by the head of department CMC and/or Amsterdam UMC QA manager, the Amsterdam UMC board of directors is consulted, in order to define an appropriate corrective and preventive action plan.

8. Approval form

The principal investigator (PI) or delegate will permit monitoring and make time available to meet with the CRA on a regular basis to discuss the progress of the study. In case of non-response from the PI of a participating site, the coordinating PI or delegate is contacted.

The coordinating PI will only delegate study related tasks to qualified study staff members.

Furthermore, the coordinating PI or delegate confirms that read only and verification access to the eCRF is permitted for the CRA allocated to the study.

By signing this monitoring plan the coordinating PI agrees upon following the laws and regulatory requirements, e.g. WMO, ICH-GCP and/or ISO14155, and take appropriate measures in case of high priority monitoring issues, recurrent protocol deviations and/or serious breaches.

I have read this monitoring plan and agree with content outlined in the document.

Coordinating principal investigator: Prof. R.M. van den Berg-Vos, MD, PhD

Signature Date 03-04-2025

Sponsor's head of department: Prof. D. van de Beek, MD, PhD

Signature Date 15-04-2025

Appendix I: Types of monitoring

Site initiation visit
The objective of a site initiation visit is to train the study staff members in the study protocol and in the relevant study related procedures. A site initiation visit is performed after approval is obtained applicable regulatory authorities and preferably also from the local board of directors and before initiation of the enrolment of study subjects.

A site initiation visit is required for each participating site, including the sponsor site. In general, site initiations visit(s) are arranged and performed by the coordinating PI or delegate of the sponsor site. For all participating sites, it is expected that, at a minimum, the local PI will attend the site initiation visit. Additionally, involved study coordinators, co-investigators and other study staff members are recommended to attend the site initiation visit. The site initiation visits can be replaced by a centralised initiation meeting.

A site initiation training log or attendance log is completed during the site initiation visit or centralised initiation meeting, enlisting all the study staff members present. In case of a site initiation visit, the original log is maintained on site, in the ISF; a copy of the log is filed at the sponsor site in the TMF. In case of a centralised initiation meeting the original log is maintained at the sponsor site in the TMF; a copy of the log is filed at each participating site. If a member of the study team cannot attend the site initiation visit or centralised initiation meeting, the study staff member is trained by the PI or a delegated member of the study team who attended the site initiation visit or centralised initiation meeting. This training is documented on a local study training log and filed in the ISF.

On-site monitoring visit
The objective of an on-site monitoring visits is to ensure that all study procedures are correctly performed and adhered to, all required study data is entered in the (e)CRF and is verifiable in the source documents and to identify any issues that the site may encounter with the enrolment of eligible study subjects. On-site monitoring visits are performed on a regular basis, following site initiation until last study subject last visit and finalisation of study data entry in the (e)CRF.

On-site monitoring visits are required for each participating site, including the sponsor site and are scheduled based on the current NFU-guideline '*Kwaliteitsborging van Mensgebonden Onderzoek*'. On-site monitoring visits are performed by the CRA allocated to the study. A specification of the activities that are performed by the CRA during an on-site monitoring visit is specified in section 6 of this monitoring plan.

Remote monitoring
Remote monitoring is utilised by the CRA as a method of maintaining oversight of a study or a participating site, in between the routine on-site monitoring visits. Remote monitoring includes the request for regular status updates from the coordinating PI or delegate on e.g. study subject enrolment and study protocol amendments, as well as regular communication with the participating site(s) on operational issues such as site staff changes and the resolution of monitoring issues.

In addition remote monitoring may include central monitoring, which consists of remote review of the eCRF or remote verification of the eTMF. Centralised monitoring will be restricted to the types of information that are available and/or accessible to the CRA on a remote basis, ensuring compliance to the current privacy legislation.

Site close out
The objective of a site close out is to ensure all monitoring activities are completed, e.g. verification that all study data is collected and all outstanding queries are resolved and closed, all essential documents are available and filed in the TMF/ISF or ISF, all monitoring issues are resolved or may be considered closed. In addition, the site to be closed is informed on the requirements related to archiving of the study documents, possible inspections and other applicable long term requirements related to the conduct of a clinical study.

Site close outs are required for each participating site, including the sponsor site. A site close out is conducted by means of an on-site close out visit or by means of a remote site close out. The type of site close out applicable to a study is specified in section 5 of this monitoring plan.

In case of an on-site close out visit, the visit is performed when all study subjects enrolled at the site to be closed have completed the study and all study data is collected and entered in the (e)CRF, though prior to database lock, or following premature closing of the site. On-site close out visits are performed by the CRA allocated to the study.

In case of a remote site close out, the close out is initiated when all study subjects enrolled have completed the study, all study is collected and entered in the (e)CRF and in general, after database lock, or following premature closing of the site. A remote site close out is initiated by the CRA allocated to the study. The PI of the site to be closed receives a customised remote close out checklist per e-mail, listing all applicable site close out activities. The PI or delegate of the site to be closed returns a copy the completed checklist to the CRA, signed and dated by the PI of the site to be closed, to confirm the study is adequately closed.

Appendix II: Participating sites

Participating site	Amount of planned study subjects	Name contact person and role	Contact details: phone/e-mail
Amsterdam UMC	25	P. Nederkoorn, MD	p.j.nederkoorn@amsterdamumc.nl
Catharina Ziekenhuis	25	R. Gons, MD	rob.gons@catharinaziekenhuis.nl
Elisabeth-TweeSteden Ziekenhuis	24	B. Jansen, MD	b.jansen@etz.nl
Elkerliek Ziekenhuis	23		
Flevoziekenhuis	14	E. Osei, MD	losei@flevoziekenhuis.nl
Franciscus Gasthuis en Vlietland	39	K. Dorresteijn, MD	k.dorresteijn@Franciscus.nl
Gelre Ziekenhuizen	24		
Haaglanden MC	24		
Isala Klinieken	36	W. Jolink, MD	w.m.t.jolink@isala.nl
Jeroen Bosch Ziekenhuis	24	M. van Zagten, MD	m.v.zagten@jbz.nl
Leids UMC	36		
Maasstad Ziekenhuis	36	W. Moudrous, MD	MoudrousW@maasstadziekenhuis.nl
Maastricht UMC	14	J. Staals, MD	j.staals@mumc.nl
Medisch Centrum Leeuwarden	36		
Medisch Spectrum Twente	23	R. Arntz, MD	renate.arntz@mst.nl
OLVG	25	Prof. R. van den Berg-Vos, MD	r.vandenberg-vos@olvg.nl
Rijnstate ziekenhuis Arnhem	25	S. Vermeer, MD	svermeer@rijnstate.nl
Sint Antonius Zkh, Nieuwegein	36		
Tergooi MC	24		
UMC Groningen	24		
UMC Utrecht	13		

Viecuri MC	13	F. de Kleermaeker, MD	fdkleermaeker@viecuri.nl
Zuyderland Medisch Centrum	7	T. Schreuder, MD	t.schreuder@zuyderland.nl
