# Supplementary material for: Rationale and design of ‘StAtins in Frail oldEr patients with ischemic Stroke or Transient ischemic attack–the Randomized Controlled Trial’ (SAFEST-RCT)
Source: BMJ Neurol Open. 2025 Oct 5;7(2):e001297. doi: 10.1136/bmjno-2025-001297 (PMC12506154; doi:10.1136/bmjno-2025-001297)
Supplement: online supplemental file 4 [file bmjno-7-2-s004.docx]

Data management

| The following study info provides relevant information regarding data management requirements. We included two related projects: SAFEST RCT and SAFEST cohort. The data collection and data management is similar for both research projects. | |
| --- | --- |
| Study number (ABR/METC) | SAFEST cohort: 2024.0202 (METC)  SAFEST RCT: 86273 (ABR) |
| Study (acronym or short title) | SAFEST-cohort and SAFEST-RCT |
| Sponsor (Verrichter/initiator/coördinerende hoofdonderzoeker) | 1. Prof. dr. Renske van den Berg-Vos  2. Prof. dr. Nathalie van der Velde |
| Department | 1. Neurologie  2. Interne geneeskunde, sectie geriatrie |
| Mono/Multicenter & (inter)national | Nationaal multicenter |
| (non)WMO | WMO compliant (SAFEST RCT)  not WMO compliant (SAFEST cohort) |
| Summary of the project | The SAFEST-RCT is a Multicenter Prospective Randomized open-label study with blinded evaluation of endpoints (PROBE). The aim is to assess the impact of starting versus not starting a statin in frail individuals aged 70 and above with a recent ischemic stroke or transient ischemic attack (TIA) on their health-related quality of life (hrQoL) and major adverse cardiovascular events (MACE)-free survival during a two-year follow-up period.  The SAFEST-cohort is a prospective cohort study. The aim is to assess the impact of deprescribing versus continuing statin therapy in frail individuals aged 70 and above with a recent ischemic stroke or transient ischemic attack (TIA) on their health-related quality of life (hrQoL) and major adverse cardiovascular events (MACE)-free survival during a two-year follow-up period.  We will include 300 participants in the SAFEST cohort and 600 participants in the SAFEST RCT, from 24 Dutch Hospitals. |
| Co-ordinating PI (Hoofdonderzoeker) | Prof. dr. Renske van den Berg-Vos |
| Responsible for completion of the  data management plan | Susanna Prins and Birgit Damoiseaux-Volman |
| Funding by | Evaluatieonderzoek ZE&GG, ZE&GG Ronde 2020, ZonMw dossiernummer 10330032010007 d.d.21/01/2022 |
| Consulted data management expert | *Meriem Manaï, Research Data Management consultant/ontwikkelaar, m.manai@amsterdamumc.nl, 30-05-2022 & 03-06-2024* |
| Data management plan version / date | Version 2 / 11-06-2024 |
| PI signature for approval of this DMP | 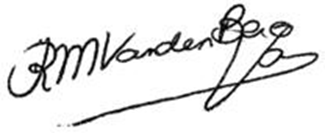 |

| **Phase 1: Study preparation** | |
| --- | --- |
| **Privacy and security safeguards** | |
| **1.1** | The data set is anonymous and cannot be linked to any subject  The data set is encoded; a meaningless unique code (Subject ID) is used, and subjects can be identified through a subject identification log. Data are encoded (by assigning participant identification (ID) numbers to all included patients) to ensure the security of privacy-sensitive information and/or to get in touch with subjects during data collection. The assigned participant ID numbers are subsequently used to merge data that are collected over multiple time points and from multiple sources. The participant ID number also allows future additions to the data.  The data set is directly identifiable*.* |
| **1.2** | Data are de-identified within the study database (see 1.1)  Additional de-identification steps are taken. |
| **1.3** | A Data Protection Impact Assessment (DPIA) has been performed and stored in the digital archive ( (for location – see 1.27)  The data acquisition has been registered at the DPO, *Verwerkingsregister number 138235 for both SAFEST-cohort and SAFEST-RCT* |
| **1.4** | The study has been (pre)registered or a concept/design paper has been published: *Both SAFEST-RCT and SAFEST cohort are registered in the clinicaltrials.gov trial register (number 2022-502059-79-00 and number …., respectively). A design paper for SAFEST RCT is being written and will be published.* |
| **1.5** | An informed consent procedure has been set up that describes the data set, time span of data retention, information on sharing data or making data available for future research. |
| **1.6** | A central location for all digital study documents and (references) to data exists and is stored in the digital archive ( (for location – see 1.27)   A central location for all hard copy study documents exists. For location, see 1.27 |
| **Data acquisition** | |
| ***General*** | |
| **1.7** | Describe the data acquisition per type of data  Reuse of existing data: this refers to using patient characteristics and other data from the electronic patient dossier, and includes:   - *Age (in years)* - *Sex (male, female, other)* - *Type of index event*   - *Ischemic stroke or TIA*   - *NIHSS (0 – 42)*   - *Trial of Org 10172 in Acute Stroke Treatment (TOAST) classification: LAA (Large-Artery Atherosclerosis), CE (Cardio-Embolic), SVO (Small-Vessel Occlusion), Other Determined Etiology, Undetermined Etiology.* - *History of cardiovascular events, using ICD-9 and ICD-10 codes* - *Charlson Comorbidity index (0-37)* - *Fall history (number of times in the last 12 months)* - *Risk factors for cardiovascular events*    - *Smoking: never, former, current*   - *Alcohol:*   - *Premature cardiovascular disease in the family: yes, no, unknown* - *Medication use (ATC codes)* - *Length (in cm)* - *Weight (in kg)* - *Systolic blood pressure (mmHg)* - *Diastolic blood pressure (mmHg)* - *Glucose (mmol/L)* - *HbA1c (%)* - *Creatinine (umol/L)* - *eGFR (mL/min/1.73 m²)* - *ASAT (U/L)* - *ALAT (U/L)* - *Total cholesterol (mmol/L)* - *LDL cholesterol (mmol/L)* - *HDL cholesterol (mmol/L)* - *Triglycerides (mmol/L)* - *Contact information of the participant*   Use of measured data:  Data collection*:*   - *Health-related Quality of Life (hrQoL) measured with the PROMIS-10 Global Health questionnaire* - *3-point Major Adverse Cardiovascular Events (MACE) including cardiovascular death, nonfatal myocardial infarction and nonfatal stroke.* - *Non-cardiovascular death* - *Frailty, assessed using the Clinical Frailty Scale (CFS)* - *Functional outcome assessed using the modified Rankin Scale (mRS)* - *Cognitive outcome measured using the Montreal Cognitive Assessment (MoCA) or the Telephone version of the MoCA (T-MOCA)* - *Cost-effectiveness and societal costs assessed using the EuroQol five-dimension 5 level questionnaire (EQ-5D-5L) and The Older Persons and Informal Caregivers Survey – Minimum DataSet (TOPICS-MDS) question 25-37* - *Falls (number of falls, date) using a calendar* |
| **1.8** | Describe the terminology standards, classifications or existing data definitions that have been applied in the data set:  *See 1.7 (validated questionnaires, ATC, ICD-9/10)* |
| **1.9** | All acquired data, either reused, measured, or manually collected, are described in a data dictionary and stored in the digital archive ( (for location – see 1.27)*.* |
| **1.10** | \| Data acquisition type \| Description \| Type \| Format \| Size estimate \| Sensitivity \| Terminology standard \| \| --- \| --- \| --- \| --- \| --- \| --- \| --- \| \| *[1. Descriptive documents,*  *2. Reuse data,*  *3. Measured data,*  *4. Collected data,*  *5. Analysis data,*  *6. Publication or archiving (meta)data]* \| *[e.g., TMF/ISF or separate Protocol, ICF, agreements, etc.;*  *Data dictionary/codebook,*  *Raw questionnaire data, interview recordings, etc.]* \| *[e.g., images, scans, spreadsheet, script]* \| *[e.g., .csv, .dicom, .sav, .pdf. odt, .jpg, .sps, .mxf, .rdf, .xml, REFI-QDA, etc.]* \| *[e.g., ~1MB, <1GB, xx GB, ~1TB, mention total per type of file format expected]* \| *[1. Highly sensitive personal data (interview data/directly identifiable data),*  *2. Medium sensitive data (encoded subject data),*  *3. Low/non-sensitive data (standardized questionnaire without specific demographic or medical history data, etc.)]* \| *[e.g., ATC for type of medication, LOINC for lab values, SNOMED CT for treatment, ICD-10 for condition, medRA for medical coding, CDISC standards (e.g., SEND standard for pre-/non-clinical data, CDASH for collected clinical data, or SDTM for analysis data, etc.]* \| \| Reuse of existing data \| Copied from EPD \| Castor \| Castor \| n/a \| Highly sensitive \| See 1.7 \| \| Data collection \| Raw questionnaire data \| Either in Castor, or scanned from paper \| Castor / .pdf \| <1GB \| Low sensitive data \| See 1.7 \| \| Descriptive documents \| Protocols, ICF templates, questionnaire templates, etc \| Text \| Word.pdf \| <1GB \| Low sensitive \| n/a \| \| Descriptive documents \| Signed ICFs, subject ID log \| Text \| Word/PDF/Paper \| <1GB \| Highly sensitive \| n/a \| \| Analyses data \| Syntaxes/scripts \| Script \| .csv \| <1GB \| Low sensitive \| n/a \| |
| ***Reuse of existing data*** Not applicable | |
| **1.11** | Specify the source that is used to acquire the existing data:   - *At inclusion we will use data from the EPD hospital* - *At follow-up we will use data from the GP or other treating physician and the pharmacy (they will be contacted by phone and asked to fill in information on a form).* |
| **1.12** | The reuse of existing data for this study is covered by the subject’s informed consent |
| **1.13** | The party that delivers encoded data remains responsible for the subject identification log for their own subjects. |
| ***Measured data*** Not applicable | |
| **1.14** | *Specify the device that generates the measured data:* laboratory and clinical tests, performed by the nurses for clinical care. |
| **1.15** | No user training is required; the device that generates the data is self-explanatory  No separate user training is required; users are already acquainted with and trained in using the device for the type of measurements that are required for this study  Users are trained in using the device /performing the lab measurements in the way needed for this study this is documented |
| ***Data collection***  Not applicable | |
| **1.16** | An electronic CRF is used to collect all or a part of the data, a copy of the blank CRF pages is kept as back-up  A paper CRF is used to collect all or a part of the data |
| **1.17** | *Specify the name of the data collection system: Castor*  Licensing and a Processing agreement have been arranged |
| **1.18** | The developer of the eCRFs and/or questionnaires in the data collection system is already acquainted with the system.  The developer of the eCRFs and/or questionnaires in the data collection system is trained in using the system and this is documented. |
| **1.19** | The database was designed before it was built and a data dictionary was created, specify document name and location of the data dictionary: *see 1.9* |
| **1.20** | Validation checks on completeness, correctness and consistency are incorporated in the data collection system and have been documented*, testplan volgens trialbureau ZE&GG* |
| **1.21** | The data collection system has been tested by both the study team and an independent party.  *Study team:: 31012023 by Birgit Damoiseaux-Volman, Postdoc*  *Independent party: 31012023 by Annelies Rotte (consulent & owner Trial data solutions)*  The test findings, follow-up of the findings and final approval are documented, *specify document name and location:*  *Will be placed at sharepoint when finalized.* |
| **1.22** | Access to the data collection system is based on individual login with only the necessary access rights  Access to the data collection system is managed (under supervision of) and documented by the PI |
| **1.23** | The data collection system logs the identity of the persons using the system |
| **1.24** | Procedures for data collection are included in the data collection system  Procedures for data collection are documented in a manual, *specify document name and location: …* |
| **1.25** | No user training is required; the data collection system is self-explanatory  No user training is required; users are already familiar with the data collection system (only non-WMO)  Users are trained in the data collection system, and this is documented. *A Standard Operating Procedure is made and stored in the digital archive ( (for location – see 1.27).* |
| **1.26** | For each study-specific data collection, source documentation is available  For the following parts of data collection, no source documentation is available, as described in the protocol; *specify: information that was noted directly in Castor (see 1.9)* |
| **1.27** | What data (raw files, intermediate files, final files, subject identification log (key file)) is stored on which location during the study?   \| Location \| What data? \| \| --- \| --- \| \| On the department’s L-drive (Amsterdam UMC, location AMC computers - *start – this pc – L-drive – onderzoek – divg - SAFEST*) \| Raw  Interim  Final  Key \| \| On a data storage facility at Amsterdam UMC (*department H2, room for vascular neurology, black locked archive cabinet*) or at an Amsterdam partner (Castor) \| Raw  Interim  Final  Key \| \| On an external data storage facility; see the L drive for the list of 24 participating centres, in which pseudomication keys are stored. \| Raw  Interim  Final  Key \|   *During the data collection raw data will be stored in the data storage facility. During archiving, part of the raw data will be scanned and saved on the L-drive (see 5.13)* |
| **1.28** | The size of the data is <10 gigabyte during data collection and <10 gigabyte when archiving the project  Budget is allocated both for storage during the study as well as for archiving upon completion of the study  Budget is allocated for data management activities and creating a FAIR dataset |
| **1.29** | The study-specific folder with data files is only accessible by study team members  Access to study data is managed and documented by the PI |
| **Subject identification log**  Not applicable | |
| **1.30** | The subject identification log(s) is/are kept separate from other study related data. |
| **1.31** | In multicentre studies the site-specific subject identification log(s) is/are kept on site only and will not be shared centrally or with other sites. *However, to be able to contact the participants for follow-up, the contact information of the participants will be collected and shared with the investigators at Amsterdam UMC via an independent, secondary database in Castor.* |
| **Legal issues/agreements/matters**  Not applicable | |
| **1.32** | For collaboration with the research partners or for data transfer or data sharing, written agreements on data management, privacy, data ownership and intellectual properties are made, namely:  A clinical trial agreement with the participating centres. This is stored in the digital archive ( (for location – see 1.27)*.*  An agreement with other parties, e.g., data sharing agreement or material transfer agreement, *specify: …* |
| ***Additional information on Phase 1: Study preparation (provide item number)*** | |
|  | |

| **Phase 2: Data collection** | |
| --- | --- |
| **General** | |
| **2.1** | A site signature and delegation log of all people involved in the data collection is made and is kept by the PI |
| **2.2** | *If applicable:* a procedure for deblinding is in place and has been documented |
| **Reuse of existing data / use of measured data**  Not applicable | |
| **2.3** | The reused existing data and/or the raw, measured data are stored as read-only file and a copy is made for further processing |
| **Externally acquired data**  Not applicable | |
| **2.4** | Identifiable data is removed or encoded by the external party, prior to sharing the data |
| **2.5** | Data are transferred in a secure way by SURF Filesender |
| **Quality control** | |
| **2.6** | Checks on completeness, correctness and consistency are built into the system (as specified in the *testplan volgens ZE&GG)*  Non-automated checks such as manual checks and data monitoring are performed and are documented |
| **2.7** | Completion of multicentre data collection is signed off by the local PI per patient and the coordinating PI for completion of data collection |
| **Change control**  Not applicable | |
| **2.8** | How are changes to the **data** handled?  Documented on the paper data collection tool (pCRF or questionnaire)  Audit trail or ‘track changes’ functionality in the applied system  Reason for change is documented, e.g., ‘Confirm changes’ setting in Castor is used  Other change control; *specify:* … |
| **2.9** | How are changes to the **design** of the data collection handled?  By creating a new version of the paper data collection tool (pCRF or questionnaire)  Audit trail or ‘track changes’ functionality in the system  Other change control procedures; *specify:* …  All changes in the design will be documented to ensure an impact assessment of these changes is performed |
| **Locking a data collection**  Not applicable | |
| **2.10** | *Describe how the data collection system is locked:*  Using the locking functionality in the system  Other; *specify:* … |
| **2.11** | Approval and reason for locking have been documented |
| **2.12** | The Statistical Analysis Plan is finalized, prior to (deblinding and) analysing the data |
| ***Additional information on Phase 2: Data collection (provide item number)*** | |
|  | |

| **Phase 3: Processing & statistical analysis** | |
| --- | --- |
| **Export to the data processing and statistical environment**  Not applicable | |
| **3.1** | The data are stored in a generic and machine actionable format; *specify: CSV*  Data are also stored in another format; *specify: RDATA, see 1.10* |
| **3.2** | *Specify the software system including version number, applied for processing and statistical analysis: R (version number 4.2.3)* |
| **Performing data processing and statistical analysis**  Not applicable | |
| **3.3** | The acquired data are stored as read-only file and a new file is created for further processing and statistical analysis |
| **3.4** | All data processing and analysis is programmed in syntax or script files |
| **3.5** | Descriptive comments are added to the syntax or script files |
| **3.6** | Data sets and syntax or script files are placed under version control |
| **3.7** | Data corrections in this phase are made in the original source  Data corrections in this phase are programmed in syntax or script files |
| **Sharing data for processing or statistical analysis**  Not applicable | |
| **3.8** | Data processing and analysis by an external party is covered by the informed consent |
| **3.9** | Data are transferred in a secure way by SURF Filesender or *specify which secure transfer is used: …* |
| ***Additional information on Phase 3: Processing & statistical analysis (provide item number)*** | |
|  | |

| **Phase 4: Writing & publishing** | |
| --- | --- |
| **Organizing files** | |
| **4.1** | For each manuscript a structured subfolder has been created. |
| **Findability of the data set** | |
| **4.2** | The manuscript will be published in an Open Access journal that provides a PID (e.g., a DOI or URN)  The information regarding the data collection can be found through a catalogue or repository; *DataverseNL*  This catalogue or repository creates a PID  This catalogue or repository has a CoreTrustSeal (or other certification; *specify: …*)  To make my (meta)data findable, we will crosslink any online sources where applicable *(e.g., ORCIDs of researchers, PIDs of related publications or repository references within the project, trial registry numbers, project website, etc.)*  I will not publish my metadata and/or data; *explain why not …* |
| ***Additional information on Phase 4: Writing & publishing (provide item number)*** | |
|  | |

| **Phase 5: Data sharing and archiving** | |
| --- | --- |
| **5.1** | What will be published?  Metadata (see 5.2)  The raw, pre-processed data  The final data. *Due to the sensitivity of the data, the data will be archived internally on the L drive (for location – see 1.27) and made available upon reasonable request.*  Other; *specify type and location: …* |
| **5.2** | What metadata is provided about the **study?**  Study protocol  (Amsterdam UMC) metadata schema  Statistical Analysis Plan  Other; *specify …*  Data Management Plan |
| **5.3** | Data reuse of the dataset is covered in the informed consent procedure  Information regarding the subset of the data for people who consented to reuse is available  Procedures for withdrawal of consent for reuse have been defined  A more pseudonymized version of the dataset has been created for reuse in consultation with the DPO  For verification purposes, all data are stored internally (see 5.5 for data access procedures). |
| **5.4** | What metadata is provided about the **data**, including processing and analysis?  Documentation on study procedures  Syntaxes or scripts  Data dictionary  Software or hardware  Data validation and derivation plan  Other; *specify: …* |
| **5.5** | The research data will be publicly accessible without any restrictions:  Conditions for reuse apply; *provide a short description: Evaluated based on the informed consent form and privacy considerations*  An embargo period applies; *specify reason and duration: …*  At the time of the journal article’s publication |
| **5.6** | For data reuse, a Data Sharing Agreement or equivalent will be set up |
| **Transfer to an external party**  Not applicable | |
| **5.7** | (Copies of) original data and documentation are kept at Amsterdam UMC  All data transfers have been documented |
| **5.8** | Data are transferred in a secure way by SURF Filesender  In case future data requests can be met and data needs to be transferred to an external party, this will be done in consultation with Legal Research Support and the Data Protection Officer/ Privacy experts, to ensure the appropriate agreement is in place, and data will be transferred in a secure way, e.g. by using SURF filesender. |
| **Digital archiving** | |
| **5.9** | *Digital data and documentation will be preserved for 25 years.* *This includes:*  metadata  raw data files  final data files  *If any of these boxes is not ticked, explain: …* |
| **5.10** | *Specify the location of the digital archive: see 1.27* |
| **5.11** | A subject identification log is archived in the digital archive and kept separate from other study related data. This does not conflict with the subject’s informed consent. |
| **Paper archiving**  Not applicable | |
| **5.12** | *Paper documentation will be preserved for 25 years* |
| **5.13** | *Specify the physical location of the paper archive: - ICFs, site signature & delegation log: on paper, stored in a data storage facility at Amsterdam UMC (for location – see 1.27) - Raw data from questionnaires: scanned and documented in the digital archive (for location – see 1.27)* |
| ***Additional information on Phase 5: Data sharing & archiving (provide item number)*** | |
|  | |
